# African Swine Fever in Wild Boar: German Hunters’ Perception of Surveillance and Control—A Questionnaire Study

**DOI:** 10.3390/ani13182813

**Published:** 2023-09-05

**Authors:** Lisa Rogoll, Katja Schulz, Franz J. Conraths, Carola Sauter-Louis

**Affiliations:** Friedrich-Loeffler-Institut, Federal Research Institute for Animal Health, Institute of Epidemiology, Südufer 10, 17493 Greifswald-Insel Riems, Germany; katja.schulz@fli.de (K.S.); franz.conraths@fli.de (F.J.C.); carola.sauter-louis@fli.de (C.S.-L.)

**Keywords:** African swine fever, wild boar, hunters, participation, surveillance, control

## Abstract

**Simple Summary:**

Effective control of African swine fever in wild boar relies on cooperation with hunters, who are involved in the local implementation of surveillance and control measures. This study focused on understanding German hunters’ perceptions of different control measures and factors that influence compliance. Measures that hindered hunting were generally considered ineffective. Some measures were seen as controversial as they were seen as contrary to fair hunting practices. Effective communication and raising awareness are recommended to improve compliance with controversial measures. This study also highlighted the need to address hunters’ concerns and provide adequate compensation to maintain their motivation to participate in ASF control efforts. Among others, financial incentives and reduced bureaucracy were identified as motivating factors.

**Abstract:**

Since the first occurrence of African swine fever (ASF) in wild boar in Germany in 2020, the disease has primarily affected the wild boar population in the eastern part of the country close to the border with Poland. Local hunters play a crucial role in implementing surveillance and control. To evaluate their perceptions of existing control measures and analyze regional differences between hunters from ASF-affected and non-affected regions, a questionnaire study was conducted among the German hunting community. Hunters from non-affected areas held a more optimistic view regarding the effectiveness of control measures compared to hunters from affected areas. However, control measures that hinder hunting were generally perceived as ineffective. Measures that collided with hunters’ understanding of fair hunting practices were regarded as controversial. Financial incentives and reducing bureaucracy were the most favored approaches to increase hunters’ participation. Moreover, the possibility of eating or selling the meat of hunted wild boar and the provision of infrastructure for implementing ASF control were considered motivating. Thus, this study highlights the importance of compensating hunters and addressing their concerns to maintain their engagement in ASF control. To enhance compliance with controversial measures, thoughtful communication and raising awareness are essential.

## 1. Introduction

African swine fever (ASF) is a severe hemorrhagic disease of different suid species, including domestic pigs and wild boar. The disease originates from the African continent and can cause a variation of symptoms, ranging from peracute death to subclinical infection [1]. The disease has been absent from the European continent since 1995 with the exception of the Italian island of Sardinia, where ASF was endemic from 1978 to recently [2]. The virus that currently circulates in Eastern, Southern, and Central Europe was introduced into Georgia in 2007 [3]. In the following years, ASF spread throughout the Caucasus Region and reached the Russian Federation, where it mainly affected domestic pigs [4]. Subsequently, the disease was introduced into the European Union, where it was first discovered in Lithuania in January 2014 and Poland one month later [5]. It then spread to Latvia in June 2014 [6] and Estonia in September 2014 [7]. The disease was subsequently introduced into several other countries including the Czech Republic, Romania, Belgium, Bulgaria, Greece, Hungary, Serbia, and Slovakia [8]. In November 2019, ASF unexpectedly emerged in wild boar in Western Poland [9]. Ten months later, on the 10th of September, the first case of ASF in wild boar in Germany was confirmed close to the Polish border [10]. Meanwhile, several other countries became affected by ASF, such as Bosnia and Herzegovina, Croatia, and the Italian mainland (outside of Sardinia).

The ongoing westward spread of the virus throughout the European Union is mainly driven by virus persistence in wild boar populations, described as the “wild boar habitat cycle” [11]. The virus can be transmitted from infected wild boar or contaminated carcasses to susceptible conspecifics [11]. In addition, humans represent a risk factor for the long-distance transmission of ASF and its introduction into domestic pig holdings [12]. The lasting presence of the ASF virus in wild boar populations poses a risk of infection for commercial domestic pig holdings and leads to trade restrictions that cause huge economic losses [13].

By July 2022, wild boar populations in three out of the sixteen federal states of Germany were affected by ASF: Saxony, Brandenburg, and Mecklenburg–Western Pomerania (as reported to the EU Animal Disease Notification System). The disease has apparently been eliminated in the affected wild boar population in Mecklenburg–Western Pomerania, and control seems to be successful in large parts of Brandenburg [14,15]. However, there are still cases emerging in Brandenburg and Saxony. So far, eight outbreaks in domestic pig farms occurred in the German federal states of Brandenburg, Mecklenburg–Western Pomerania, Saxony, Lower Saxony, and Baden–Wuerttemberg. 

Mainly, passive surveillance is used for ASF detection in wild boar in Germany [10]. The importance of passive surveillance (i.e., searching, sampling, and removing wild boar carcasses) for the detection of ASF cases has been proven crucial by several studies showing that the probability of finding ASF-positive animals is much higher in animals found dead compared to hunted animals [16,17]. On the other hand, active surveillance (i.e., sampling apparently healthy wild boar through hunting) and increased hunting of wild boar to reduce the susceptible population and decrease wild boar reproduction rates is another important part of ASF control [18,19]. 

Regarding the implementation of the above-mentioned ASF control measures in wild boar, local hunters represent one of the most important stakeholders. Their willingness to participate in the implementation of measures such as intensified hunting, the search for wild boar carcasses, the sampling of dead wild boar, and increased biosecurity is of utmost importance for the success of surveillance and control measures. In addition, their knowledge of the local situation, in particular the wild boar population, is an important basis for the planning and implementation of measures. Thus, the necessity for research about hunters’ perceptions of ASF control has been highlighted [20,21,22,23]. Different approaches have recently been used, e.g., performing participatory studies with the hunting communities of Latvia and Estonia [24,25] and conducting a questionnaire and a participatory study in Lithuania [26,27].

Building on these studies, we aimed to capture German hunters’ perception of ASF surveillance and control in the German wild boar population by performing a web-based questionnaire study. To this end, we aimed to answer the following questions:

1. Which tasks do hunters fulfill in ASF surveillance and control and how do they assess the effectiveness of these tasks?
2. Which obstacles do hunters experience or expect when participating in ASF surveillance and control?
3. Which options do hunters consider as motivational to increase their participation in the intensified hunting of wild boar as well as intensified search for wild boar carcasses?

Based on the analysis of hunters’ replies, starting points for optimizing surveillance and control of ASF in wild boar in Germany in cooperation with the national hunting community were identified. 

## 2. Materials and Methods

### 2.1. Development and Content of the Questionnaire 

The questionnaire was designed in the German language with the web-based survey tool “SoSci Survey” (https://www.soscisurvey.de/, accessed on 15 July 2022). The final draft of the questionnaire was pretested by four hunters in the first round and six hunters in the second round evaluating the clarity of questions and response options, the length of the questionnaire, and its technical implementation. After each round, improvements and corrections were made based on the comments of the testers. 

The final version of the questionnaire (Supplementary Materials Document S1 and S2) included 26 questions that were estimated to take less than 15 min to answer. The questionnaire was divided into the four following parts:

1. Hunters’ part in ASF control and surveillance measures (questions 1 to 4 and 8 to 11);
2. Hunters’ perceptions regarding ASF control and surveillance (questions 5 to 7);
3. Motivational options for increased participation (questions 16 and 17);
4. General information about the participants (questions 12 to 15 and 18 to 26).

The questionnaire consisted of mandatory closed single-choice questions, closed and semi-closed multiple-choice questions, and three assessments based on five-point Likert scales. Voluntary open questions with the possibility of free-text input were included to allow participants to express the background of their decisions and to add suggestions to our proposed answer options.

At the end of the questionnaire, the participants also had the chance to add further comments on their perception of ASF control and surveillance. In addition, an option to submit contact data separately was implemented at the end of the questionnaire, if participants were interested in information about the results of the study or information about further participatory studies. Received contact data were saved and downloaded separately from study results, and no connection between the respective responses and the contact data of the participants was feasible.

### 2.2. Distribution of the Questionnaire

The anonymous and voluntary web-based data collection was conducted in a first period of 33 days from 29 April to 31 May 2022. The link to the questionnaire was distributed mainly via (a) private networks and social media accounts of authors and pretesters, (b) social media accounts and newsletter of the national German hunting association (“*Deutscher Jagdverband e.V. (DJV)*”), and (c) social media accounts and dashboards of German hunting magazines (“*Pirsch*”, “*Wild und Hund*”, “*Deutsche Jagdzeitung*”).

Upon the request of hunting authorities, the data collection was extended by a further 40 days in a second period from 7 June to 20 July 2022, adding up to a total time of data collection of 77 days. In the second period, the link to the questionnaire was distributed via the contact networks of hunting authorities of the German federal states *(“Oberste Jagdbehörden”*).

### 2.3. Ethics

The study received ethical clearance from the Ethics Committee of the University of Greifswald, University medicine, reference BB 044/22.

Participants were informed in writing of the background and the purpose of the study on the first page of the questionnaire. By taking part in the survey, participants agreed to an anonymous use of their answers for research and publication. No financial or other kind of compensation was rewarded for the participation. Since it was a web-based survey without any conditions of participation, everybody with access to the active questionnaire link could participate. 

### 2.4. Questionnaire Analysis 

**Figure 1:**
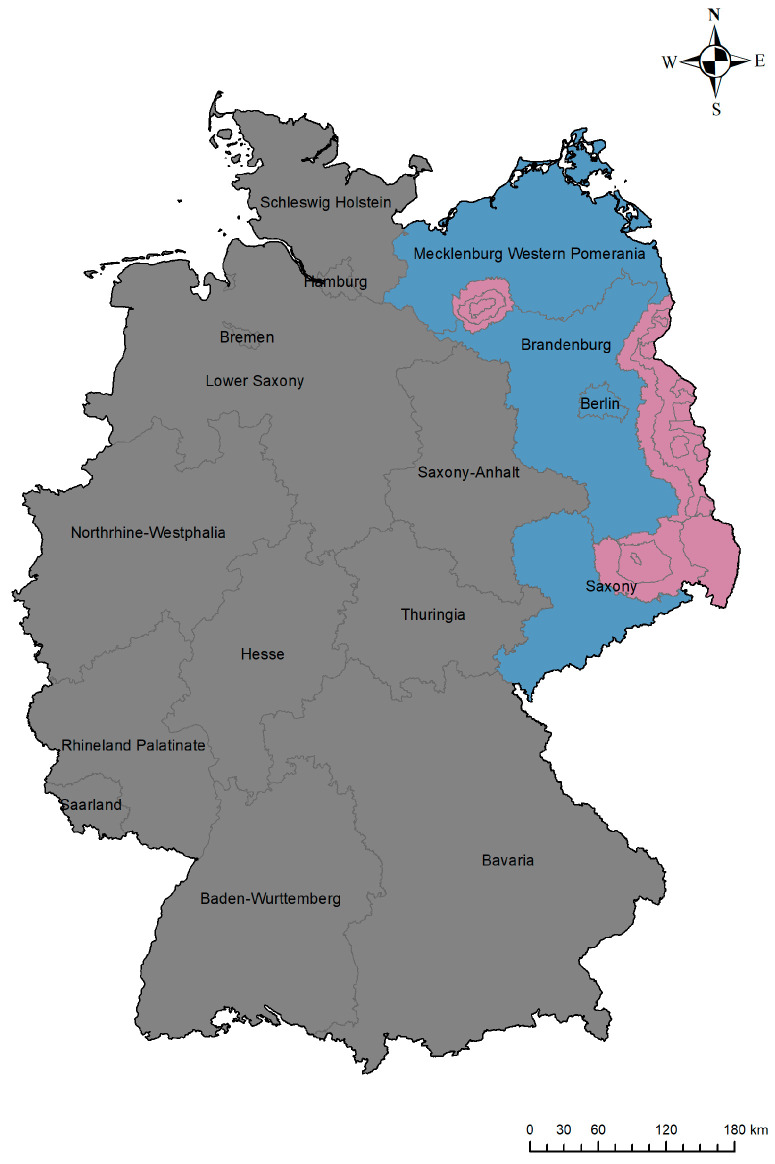
Overview of the study area. The map of Germany shows the ASF-restriction zones (red) as of 3 May 2022 based on the occurrence of ASF in wild boar (“affected”). The blue area represents the remaining areas of the federal states in the vicinity of ASF outbreaks (“vicinity”). The grey area represents federal states not affected by ASF in wild boar (“non-affected”).

For the analysis, only completed interviews with responses to all mandatory questions were considered. The data set was divided into three regional groups (Figure 1). The first group (a) (“affected”) consisted of participants who stated to hunt mainly in areas (based on postal code) that were affected by ASF and considered as restriction zones as of 3 May 2022. The second group (b) (“vicinity”) consisted of participants who hunted in at least one of the federal states neighboring the restriction zones with ASF outbreaks in wild boar. The third group (c) (“non-affected”) consisted of participants who stated neither to hunt in affected federal states nor in any state adjacent to an affected federal state (Figure 1).

Statistical analyses were conducted using the statistic software R version 4.1.2 [28]. The packages “tidyverse” [29] and “dplyr” [30] were used for data management and descriptive statistics. Graphics were created with the package “gglplot2” [31].

For the analysis of single-choice and multiple-choice questions, the relative frequencies of the answers were calculated. The results were tested for statistical differences between the three groups using the chi-squared test. After applying the Bonferroni correction, *p*-values below 0.017 were considered statistically significant.

For the analysis of answers to Likert scale questions, the relative frequencies of each level of the scale (from 1 to 5) and the median level of all answers were calculated. The results were tested for statistical differences between each of the three groups using the pairwise Wilcoxon rank-sum test with the Bonferroni correction. *P*-values below 0.05 were considered statistically significant.

Free-text responses were manually analyzed by the authors using ATLAS.ti 22 [32]. Coding was used to categorize the contents of the free-text responses systematically in order to identify patterns and themes. The frequencies for each code were counted for hunters from areas affected by ASF, hunters in the vicinity of ASF, and hunters from non-affected areas, and relative frequencies were calculated for each code in relation to the total number of free-text responses for that question. We show the top three codes for each question. The complete list of code books, code explanations, and code frequencies for each free-text question can be found in the supplementary materials.

## 3. Results

### 3.1. Response and Dropout Rates

**Figure 2:**
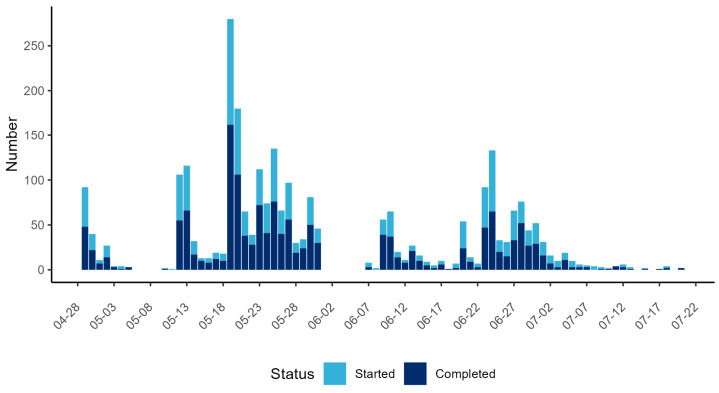
The number of started (light blue) and fully completed (dark blue) responses in a web-based questionnaire for German hunters for each day of the study period. The *x*-axis represents the date. The *y*-axis represents the number of responses received per day.

In total, 2707 filled-in questionnaires were received, out of which 1553 were complete (57%), i.e., they contained answers to all mandatory questions. Only these responses were used for further analysis. The majority of complete responses were received in the first period of data collection (*n* = 1019) (Figure 2). The distribution of the weblink to the questionnaire via the dashboards and social media accounts of hunting magazines led to the highest daily number of responses on 19 and 20 May 2022. No responses were received from 6 May to 9 May, on 14 July, 16 July, and from 19 July to 20 July 2022. From 1 June to 6 June 2022, the link to the questionnaire was offline; therefore, responses could not be received. 

More than half of the participants who did not finish the questionnaire dropped out on the second page that contained the first set of questions on hunters’ part in ASF control and surveillance measures (questions 1 and 2). An additional 17% and 10% left the questionnaire on page 3 (questions 3 and 4) and page 4 (questions 5 and 6), respectively. Only a few participants (<5%) terminated the questionnaire in other parts, except for page six, where another 8% left the questionnaire. Page six contained questions on implemented measures (questions 8 and 9).

### 3.2. General Information about Participants

The demographic distribution of the participants is shown in Table 1. The majority of the participants were male, over 40 years old, and had more than ten years of hunting experience. Regarding their hunting area, 83.9% of the participants selected only one federal state, 10.4% selected two federal states, and only 2.1% selected more than three federal states. Moreover, 1,391 hunters (89.6%) submitted the postal code of their main hunting area. Based on the postal codes, 414 participants (26.7%) hunted mainly in ASF restriction zones as of 3 May 2022, and were considered as “affected”. A number of 457 participants (29.4%) hunted in Brandenburg, Mecklenburg–Western Pomerania, and Saxony outside the restriction zones and in the City of Berlin (a separate federal state, surrounded by Brandenburg) and were considered “in the vicinity of ASF”. Another 682 participants (43.9%) hunted in federal states that have so far not been affected by ASF in wild boar and were, therefore, considered “non-affected”.

The majority of participants were owners of walk-in certificates (“*Begehungsscheininhaber*”) for hunting districts (48.9%) or leased hunting districts (46.6%). Only 6.0% of the participants owned a hunting district themselves. While 2.9% of hunters provided no specific answer to the question, 9.9% stated to organize hunting in a different way and gave a free-text response (Supplementary Table S1). The majority of them (47.7% of 153 free-text answers) stated to be professional hunters. Another 13.7% of the free-text answers stated to be hunting officers (*“Jagdaufseher”*) in their local districts, and 6.5% were regular or irregular guests in hunting districts. Hunters in affected areas and the vicinity went hunting for wild boar significantly more often than hunters in non-affected federal states (*p* < 0.001 and *p* = 0.014) and had a significantly greater estimated mean annual hunting bag of 20 and 22 wild boar hunted per year than hunters from non-affected areas with a mean annual hunting bag of 16 wild boar (*p* < 0.001) (Table 2).

Regarding the use of hunting tools, 38.5% of all hunters stated that they used silencers and night vision devices when hunting for wild boar. A total of 30.5% stated that they only used night vision devices. In contrast, 21.1% stated that they used neither silencers nor night vision devices.

More hunters in the affected federal states received financial compensation for hunted wild boar (a: 82.9%, b: 45.9%, c: 32.0%), sampling of wild boar (a: 51.0%, b: 35.9%, c: 25.8%), and notifications of detecting wild boar carcasses (a: 51.9%, b: 26.1%, c: 15.4%) than hunters in the vicinity of ASF and hunters in regions not affected by ASF. In total, 19.9% of the participants stated that they received no financial incentives and another 15.5% stated that they did not know if they were eligible to claim incentives.

A percentage of 12.5% of the participants stated that they also hunted in other European countries, and 18.0% reported that they had contact with domestic pig holdings on a regular basis. 

### 3.3. The Role of Hunters in ASF Surveillance and Control

#### 3.3.1. General Attitude of Hunters to the Success of ASF Control

Without any statistically significant differences between the groups (a vs. b: *p* = 0.361, b vs. c: *p* = 0.950, a vs. c: *p* = 0.451), 46.7% of the participants considered the elimination of ASF in wild boar in Germany possible. In contrast, 29.5% of the participants believed that the elimination of ASF in Germany is possible and 23.8% chose the answer option “I don’t know”.

In total, 1015 hunters used the free-text input to give reasons for their choice. A total of 657 hunters provided reasons for why they did not believe that ASF elimination is possible. The main reason was that they did not think it was possible to reduce the wild boar population to a level that allows ASF elimination, i.e., the population is too large and hunting is not possible in all areas (19.0% out of 657 free-text answers). They also pointed out that the routes of transmission of ASFV are too diverse and difficult to control, making it impossible to eliminate ASF (10.5%). In particular, they considered humans to be a key factor in the transmission of ASF through tourism, seasonal workers, immigration, or contamination of the environment with infectious food waste (16.6%). By contrast, 335 hunters provided reasons why they believed that the elimination of ASF is possible. They mainly stated that population reduction achieved through intensified hunting (28.7% out of 335 free-text answers) and the consistent enforcement and implementation of surveillance and control measures (21.8%) will lead to the elimination of ASF. Furthermore, success in other countries, such as Belgium or the Czech Republic, was seen as a good example that elimination might also be possible in Germany (17.6%). Out of 113 hunters who gave an explanation of why they were unsure whether the elimination would be possible or not, 9.7% thought that the elimination of ASF in Germany is hampered by continued infection pressure from Eastern European countries. However, 8.9% (out of 113 free-text answers) believed that slowing down the spread of ASF is possible.

Regarding the role of hunters in ASF control, without any statistically significant difference between the groups, 81.5% of the participants agreed with the statement that hunters play a crucial role in ASF control, 13.8% disagreed, and 4.8% chose “I don’t know” (a vs. b: *p* = 0.289, b vs. c: *p* = 0,330, a vs. c: *p* = 0.041).

A total of 1194 hunters used the free-text option to explain the reasons behind their responses. Of these, 976 hunters explained why they play a key role in ASF control. The majority of them (51.3% out of 976 free-text answers) considered hunters as a key player because they have the authority, knowledge, and equipment to reduce and control the wild boar population, which is an important part of ASF surveillance and control. It was pointed out by 38.6% that hunters know the local conditions best and are familiar with the behavior of wild boar in their hunting area. In addition, 18.0% felt that only the hunting community had the necessary knowledge and skills to control ASF. By contrast, 187 hunters provided reasons why they did not consider the role of hunters in ASF control as crucial. They believed that increased hunting would not lead to the elimination of ASF (15.5% out of 187 free-text answers) or that the wild boar population in Germany is too large to be controlled (12.8%). However, some of them believed that hunters are important but not exclusively responsible for the success or failure of ASF control. They emphasized the importance of the interaction between different stakeholders (14.4%). In addition, some hunters who were unsure whether they play a crucial role in ASF control in Germany (31 answers) pointed out that many transmission routes of ASF (particularly humans or mechanical vectors) cannot be controlled or contained by hunters (19.4% out of 31 free-text answers) and that ASF surveillance and control cannot be carried out by hunters alone—a variety of other stakeholders also need to be involved (16.1%).

The complete analysis of free-text answers for both questions can be found in the Supplementary Materials (Supplementary Tables S2–S7).

#### 3.3.2. Hunters’ Knowledge of ASF

Hunters from affected areas assessed their knowledge of ASF as statistically significantly better compared to hunters in the vicinity (*p* = 0.033) and hunters in areas not affected by ASF (*p* = 0.002). In detail, 80.0% of the hunters from ASF-affected regions stated to have rather good or very good knowledge compared to 75.3% of hunters in the vicinity and 72.0% of hunters from non-affected areas. Less than 2.0% of the total participants assessed their knowledge as very or rather poor.

**Figure 3:**
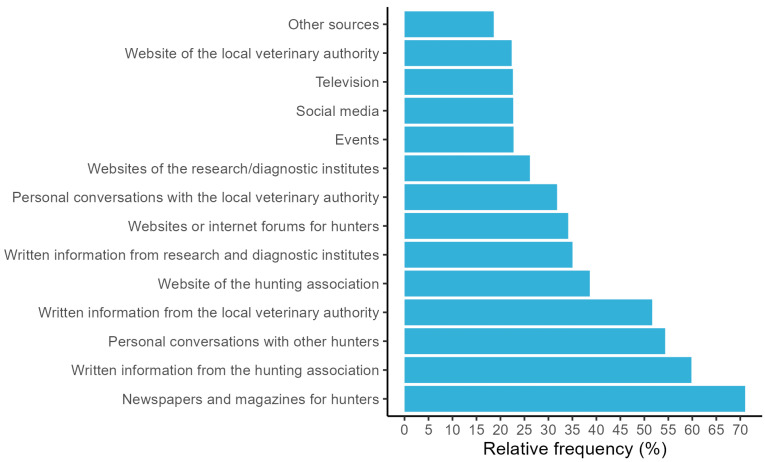
Relative frequency (in %) of hunters that used the respective source to obtain knowledge on ASF (*n* = 1553 responses to a web-based questionnaire for German hunters).

The sources for ASF knowledge that were used by more than half of the hunters were newspapers and hunting magazines (used by 71.0%), written information from the German hunting association (59.8%), personal conversations with other hunters (54.3%), and written information from the local veterinary office (51.6%) (Figure 3). The least used sources were social media (used by 22.7%), events like trade shows or lectures (22.7%), news or documentaries on television (22.6%), and websites of local veterinary authorities (22.3%). Other sources were used and explained further in a free-text input by 18.6% (*n* = 289) of the hunters (Supplementary Table S8). Of these, 24.6% stated that they acquired their knowledge about ASF in a professional context, e.g., in their professional work or during (university) studies. Another 18.4% reported that they gained their personal experience and knowledge through participation in ASF surveillance and control, and 11.8% stated that they had participated in exercises or workshops on ASF surveillance and control. 

#### 3.3.3. Surveillance and Control Measures Implemented by Hunters

**Figure 4:**
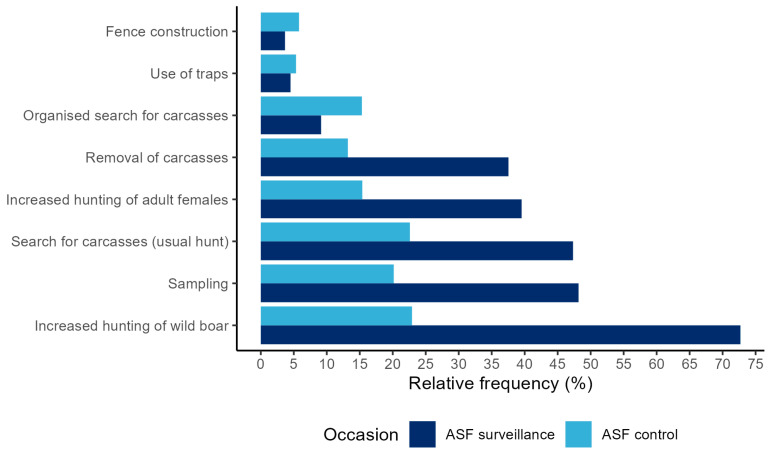
Relative frequency (in %) of hunters that implemented the respective measure in terms of ASF control or ASF surveillance (*n* = 1553 responses to a web-based questionnaire for German hunters).

As shown in Figure 4, measures were mainly implemented in terms of ASF surveillance rather than ASF control. The majority of the hunters took part in the increased hunting of wild boar (72.2%), sampling hunted wild boar that appeared sick and wild boar found dead (48.2%), and searching for carcasses as part of their usual hunting activities (47.3%) (Figure 4). The increased hunting of adult female wild boar and the removal of wild boar carcasses were carried out by 39.5% and 37.5% of the hunters as ASF surveillance measures and, to a lesser extent, ASF control measures (Figure 4).

Due to official ASF control measures, the organized search for carcasses with the help of human chains, search dogs, and drones (15.3%), the use of large boar traps (5.3%), and the construction of fences (5.8%) were used more frequently for ASF control than surveillance in the hunters’ view (Figure 4). Furthermore, 53.0% of the participants stated that they were willing to use traps in case of an ASF outbreak in their area, while 47.0% stated they were not willing to use traps.

When asked if they had implemented measures other than those listed, 391 hunters provided free-text answers (Supplementary Table S9). Of these, 16.4% stated that they had not implemented any additional measures, and 12.5% repeated options that were already mentioned in the question. The main additional measures that hunters had implemented were attending seminars or lectures on ASF (17.4% out of 391 free-text answers), training and using their own dogs to search for carcasses (11.25%), and increased participation in driven hunts or in organizing them (8.6%). 

#### 3.3.4. Assessment of Effectiveness of Measures

**Figure 5:**
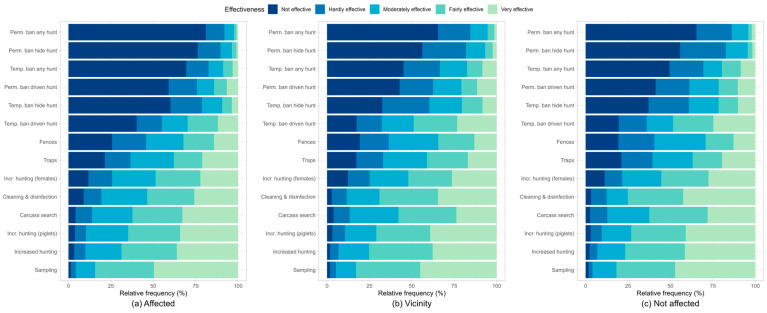
Relative frequency (in %) of hunters from ASF-affected areas (**a**), hunters from areas in the vicinity of ASF (**b**), and hunters from federal states not affected by ASF (**c**) who assessed the respective ASF control and surveillance measures in a web-based questionnaire for German hunters. The assessed measures were the sampling of wild boar hunted or found dead (sampling), increased hunting of wild boar (increased hunting), increased hunting of young animals (incr. Hunting (piglets)), an intensive search for and removal of wild boar carcasses (carcass search), cleaning and disinfection of hunting equipment/clothing and vehicles (cleaning and disinfection), increased hunting of adult females (incr. hunting (females)), use of wild boar traps (traps), construction of fences (fences), a temporary ban of driven hunts after an ASF outbreak (temp. ban driven hunt), a temporary ban of hide hunting after an ASF outbreak (temp. ban hide hunt), permanent ban of driven hunts after an ASF outbreak (perm. ban driven hunt), a temporary ban of any hunting activity after an ASF outbreak (temp. ban any hunt), a permanent ban of hide hunting after an ASF outbreak (perm. ban hide hunt), a permanent ban of any hunting activity after an ASF outbreak (perm. Ban any hunt).

The sampling of wild boar (hunted or found dead), increased hunting of wild boar, increased hunting of young animals, intensive carcass search and removal, and cleaning/disinfection were assessed as fairly effective (Figure 5 and Table 3). However, hunters from non-affected federal states assessed the increased hunting of young animals (*p* = 0.026) and intensive carcass search (*p* = 0.020) as significantly more effective compared to hunters from ASF-affected areas. Both hunters from non-affected regions and hunters in the vicinity of ASF occurrence assessed cleaning/disinfection as significantly more effective than hunters from ASF-affected areas (*p* < 0.001).

Moreover, hunters who were active in the vicinity of ASF and hunters from areas not affected by ASF considered the increased hunting of adult female wild boar as fairly effective, whereas it was assessed as moderately effective by hunters from ASF-affected regions (Table 3). Furthermore, all three groups rated the use of wild boar traps and the construction of fences as moderately effective. Hunters in regions not affected by ASF and hunters in the vicinity of ASF also assessed the temporary ban on driven hunts as moderately effective and, therefore, significantly more effective than hunters from ASF-affected regions (*p* < 0.001).

As shown in Table 3, further kinds of temporary or permanent hunting bans were assessed as hardly effective or not effective at all by all groups. However, hunters from non-affected areas and the vicinity of ASF occurrence judged the effectiveness of a permanent and temporary ban on hide hunting, a permanent ban on driven hunts, and a permanent and temporary ban on any hunting activity as significantly higher compared to hunters from ASF-affected federal states (all *p* < 0.001).

Except for the assessment of cleaning/disinfection, where non-affected hunters assessed the effectiveness as significantly better (*p* = 0.040) compared to hunters in the vicinity, no statistically significant differences were detected between both groups (Supplementary Table S10). 

The relative frequency of hunters who selected the option “I don’t know” was generally higher for every measure in the groups of hunters from areas not affected by ASF and those from the vicinity of ASF occurrence compared to hunters in ASF-affected areas (Table 3). Regarding the effectivity of the use of wild boar traps, the proportion of hunters who selected the option “I don’t know” was large compared to other control and surveillance measures (a: 13.3%, b: 16.4%, c: 19.9%). 

In total, 388 respondents provided a free-text answer to the question if they considered measures other than those already listed to be effective. Of these, 9.5% negated and 14.7% repeated measures already mentioned in the question. In addition, a variety of different measures were mentioned by the participants (Supplementary Table S11), which mainly focused on options to promote and increase wild boar hunting and raise awareness of ASF among hunters and the general public. For example, 11.6% (out of 388 free-text answers) of the participants considered the increased use of technical aids for nighttime hunting (e.g., night vision, thermal imaging, artificial light) as helpful. The payment of financial compensation to hunters or the possibility of taking paid time off work to support ASF surveillance and control measures were mentioned by 7.5% as effective measures. Raising awareness and educating the general public about ASF and its surveillance and control was mentioned by 8.5% of respondents, and a ban on entering forests after an outbreak was supported by 7.5%.

### 3.4. Hunters’ Perceptions regarding ASF Surveillance and Control 

#### 3.4.1. Consequences of ASF 

The majority of participants (66.7% out of 1553 participants) selected three to five out of the ten proposed consequences of ASF surveillance and control, regardless of the location of their hunting area.

The top two consequences, which were chosen by approximately three-quarters of the participants, were the reduction in the wild boar population in their hunting areas and increased personal work and time load (Table 4). However, hunters from ASF-affected areas selected the reduction in the wild boar population significantly more often than hunters from unaffected areas (*p* = 0.003). 

Approximately half of the participants expected or experienced local restrictions on their own hunting activity, regardless of the location of their hunting area (50.9%). However, hunters in the vicinity of ASF and hunters from non-affected areas significantly more often experienced or expected an increase in their own hunting activity (b: 56.7%, c: 58.2%) compared to ASF-affected hunters (40.6%, both *p* < 0.001). By contrast, hunters from ASF-affected areas significantly more often experienced a reduction in their own hunting activity (38.6%) compared to hunters in the vicinity (22.1%, *p* < 0.001) and hunters from non-affected regions (22.6%, *p* < 0.001).

Around one-fifth of the participating hunters expected or experienced conflicts with veterinary authorities (21.0%) and conflicts with other hunters (20.5%), regardless of the location of their hunting area. However, hunters from non-affected regions significantly more often expected or experienced conflicts with local farmers (32.1%) compared to hunters in the vicinity (23.0%, *p* = 0.001) and hunters from ASF-affected areas (17.6%, *p* < 0.001). No other statistically significant differences between hunters in the vicinity and hunters from non-affected regions were found (Supplementary Table S12). 

Significantly more hunters from ASF-affected areas experienced further consequences (14.0%) compared to hunters in the vicinity (7.7%, *p* = 0.004) and hunters from unaffected regions (8.1%, *p* = 0.002). 

A total of 148 respondents provided a free-text answer to explain further consequences (Supplementary Table S13). Of these, 18.9% reported conflicts with stakeholders other than those mentioned in the question, e.g., with forestry or animal rights activists, and in their private lives, e.g., with employers or family members. In addition, 14.9% argued that ASF surveillance and control measures, such as increased hunting or fencing, negatively affected other wildlife species, and 10.1% of respondents noted reduced or missing opportunities to sell wild boar meat and products or a price reduction for wild boar meat. Furthermore, 18.9% repeated one of the options already mentioned in the question.

#### 3.4.2. Satisfaction with Cooperation and Appreciation by other Stakeholders

**Figure A1:**
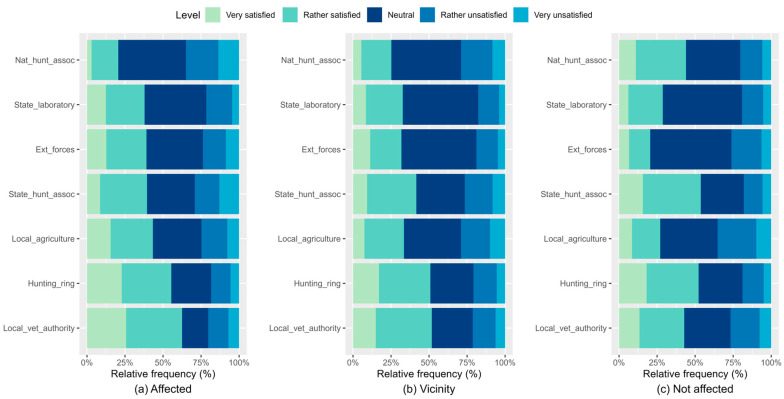
Relative frequency (in %) of hunters from ASF-affected federal states (**a**), hunters in the vicinity of ASF (**b**), and hunters from non-affected federal states (**c**) who selected the respective level of satisfaction with the cooperation with local veterinary authorities, the local hunting ring, the local agriculture, the state hunting association, external forces, the state laboratory, and the national hunting association in a web-based questionnaire for German hunters. The share of hunters who selected alternative options (“No cooperation” or “No specification”) was excluded from the analysis, as displayed in Table 5, Table 6 and Table 7.

Based on the median level of satisfaction, hunters of all three groups were “rather satisfied” with the cooperation with the local hunting ring (“*Hegering*”) (Table 5, Table 6 and Table 7). In addition, hunters from ASF-affected areas or the vicinity were “rather satisfied” with the cooperation with the local veterinary authorities (Table 5). By contrast, hunters from regions not affected by ASF were “rather satisfied” with the cooperation with the regional hunting association at the federal state level (state hunting association, “*Landesjagdverband”*) (Table 6). The satisfaction with the cooperation with other listed stakeholders was assessed as “neutral” by all three groups (Table 5, Table 6 and Table 7). However, hunters from ASF-affected regions were significantly more satisfied with the cooperation with their local veterinary authorities and the local agriculture than hunters in the vicinity of ASF (*p* = 0.003 and *p* = 0.11) and hunters from areas not affected by ASF (*p* < 0.001) (Appendix A, Figure A1, and Supplementary Table S14). Hunters in the vicinity of ASF occurrence were also more satisfied with the cooperation with the local veterinary authority compared to hunters in non-affected areas (*p* = 0.034). Furthermore, hunters from affected regions and hunters in the vicinity were significantly more satisfied with the cooperation with external forces compared to hunters from non-affected areas (*p* = 0.001 and *p* = 0.002). By contrast, hunters from areas not affected by ASF were significantly more satisfied with the cooperation with the state hunting association and the national hunting association (“*Deutscher Jagdverband e.V. (DJV)*”) than hunters from ASF-affected regions (*p* < 0.001) and hunters in the vicinity of ASF occurrence (*p* < 0.001). No statistically significant differences between the groups were detected regarding the satisfaction with the cooperation with the state laboratory (Supplementary Table S14). 

**Figure A2:**
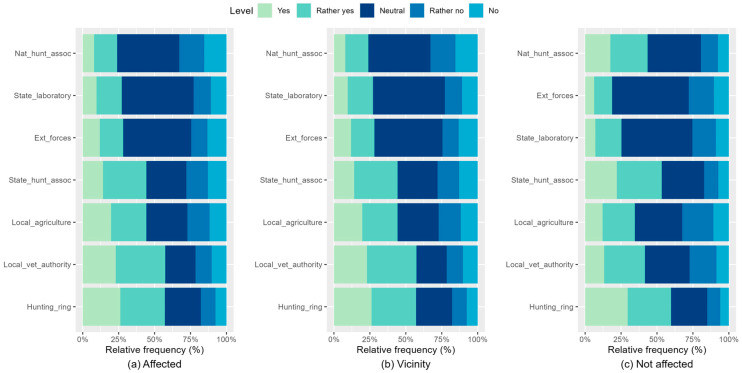
Relative frequency (in %) hunters from ASF-affected federal states (**a**), hunters in the vicinity of ASF (**b**), and hunters from non-affected federal states (**c**) who selected the respective level to rate whether they felt appreciated by the local hunting ring, the local veterinary authority, local agriculture, the state hunting association, external forces, the state laboratory, and the national hunting association in a web-based questionnaire for German hunters. The share of hunters who selected alternative options (“No cooperation” or “No specification”) was excluded from the analysis, as displayed in Table 5, Table 6 and Table 7.

Hunters of all groups felt themselves and their work were valued by their local hunting ring (Table 5, Table 6 and Table 7), although the hunters from non-affected regions felt significantly more valued compared to hunters in the vicinity of ASF (*p* = 0.040) (Appendix A, Figure A2, and Supplementary Table S15). In addition, hunters from ASF-affected areas also felt significantly more valued by the local veterinary authority compared to hunters in the vicinity (*p* = 0.004) and hunters in non-affected regions (*p* < 0.001). They also felt more valued by the local agriculture compared to hunters from unaffected areas (*p* = 0.015). By contrast, hunters from non-affected areas felt significantly more valued by the state hunting association and the national hunting association compared to hunters from ASF-affected regions (*p* < 0.001) and hunters in the vicinity (*p* < 0.001). Regarding appreciation by the state laboratory and external forces, hunters remained neutral in the median without statistically significant differences between the groups (Table 5, Table 6 and Table 7, Supplementary Table S15). 

For both questions described above, the proportion of hunters from non-affected regions who stated to have no cooperation with external forces was larger compared to hunters from ASF-affected areas (Table 5) and hunters in the vicinity (Table 6). In addition, a larger proportion of hunters from unaffected areas (Table 7) and hunters in the vicinity stated to have no cooperation with the state laboratory compared to hunters from ASF-affected regions. By contrast, a larger proportion of hunters from ASF-affected areas stated to have no cooperation with the state hunting association and the national hunting association compared to hunters in the vicinity and hunters from non-affected regions.

### 3.5. Motivational Options for Increased Participation of Hunters in ASF Surveillance and Control

Around half of the hunters (52.3% out of 1553 participants) selected three to five options, which might motivate them to increase their participation in intensified hunting in terms of ASF surveillance and control. By contrast, 4.1% of the hunters stated that none of the proposed options could motivate them. Reasons for this were given in the free text by 63 hunters (Supplementary Table S16). Of these, 22.2% stated that they already hunted as much as possible and did not have the time to increase their participation, and 19.0% considered the participation to be their duty as hunters and that no additional motivation was, therefore, needed. On the other hand, 20.6% of them did not think that an increase in the hunting of wild boar would be an effective way of controlling ASF and were, therefore, not interested in taking part.

**Figure 6:**
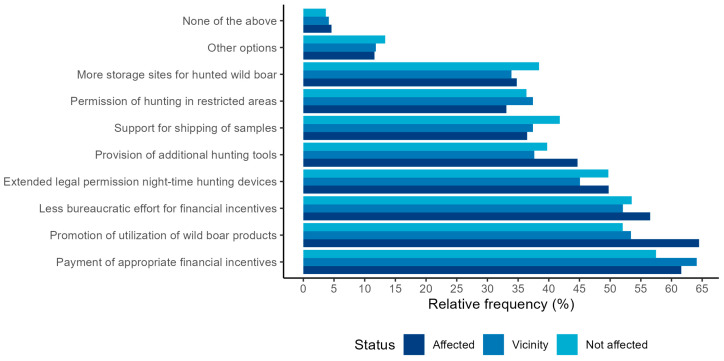
Relative frequency (in %) of hunters from ASF-affected areas (*n* = 414), hunters from areas in the vicinity of ASF (*n* = 457), and hunters from non-affected federal states (*n* = 682) who selected the respective options in a web-based questionnaire for German hunters as motivation to increase their participation in increased hunting of wild boar in terms of ASF surveillance and control.

The top three motivational options selected by more than half of hunters were the payment of appropriate financial incentives for hunted wild boar (a: 61.6%, b: 64.1%, c: 57.5%), the promotion of marketing and utilization of wild boar meat and products (a: 64.5%, b: 53.4%, c: 52.1%), and the reduction in the bureaucratic effort to receive financial incentives (a: 56.6%, b: 52.1%, c: 53.5%) (Figure 6). The proportion of hunters that selected appropriate incentives was significantly higher for hunters from ASF-affected regions compared to hunters in the vicinity (*p* = 0.001) and hunters from non-affected areas (*p* < 0.001). A noticeable proportion of hunters (a: 49.8%, b: 45.1%, c: 49.7%) also stated that an extension of the legal permission for using nighttime hunting devices would motivate them to hunt more often. In addition, the provision of additional hunting tools, such as night vision devices or silencers, was selected significantly more often by hunters from ASF-affected regions (44.7%) compared to hunters in the vicinity (36.6%, *p* = 0.041). No further statistically significant differences between the choices of the three groups were detected (Supplementary Table S17). However, a considerably larger proportion of hunters from non-affected regions considered an increase in the number of collection points for samples and support for shipping of samples (41.8%) and an increase in the number of storage sites for hunted wild boar (38.4%) as motivational compared to hunters from ASF-affected areas (36.5% and 34.8%) and those in the vicinity (37.4% and 33.9%).

Another 12.4% of the hunters stated that other options could motivate them to increase their participation. A total of 193 respondents proposed a number of different ideas, with a particular focus on options to facilitate wild boar hunting and compensate hunters for the increased workload and costs (Supplementary Table S18). The three most frequently mentioned options were the possibility of taking paid time off work to enable participation in ASF surveillance and control (8.8%), the provision of public facilities (e.g., confiscate bins) for free and the professional disposal of waste (8.3%), and changes in the hunting legislation to create more flexible hunting opportunities and changes in the system of how hunting districts are organized in Germany (7.8%).

Regarding motivational options for increased participation in carcass search, the majority of the hunters (64.0%) selected one to three of the proposed options. The total proportion of the hunters who stated that none of the listed options would motivate them to increase their participation in carcass searching and sampling (10.5%) was larger compared to motivational options for increased hunting, although the reasons were similar. A total of 163 participants explained the reasons for this in the free-text answers (Supplementary Table S19). Of these, 28.8% stated that they already participated in the carcass search as much as possible and did not have the time to increase their participation even further, and 17.8% considered participation to be their duty as hunters and that they did not need additional motivation. A further 17.2% stated that their region was not affected by ASF and that there was currently no need for carcass searching and sampling. Hunters from ASF-affected regions (13.8%) were significantly more often selected that none of the listed options could motivate them compared to hunters in the vicinity of ASF (8.1%, *p* = 0.010).

**Figure 7:**
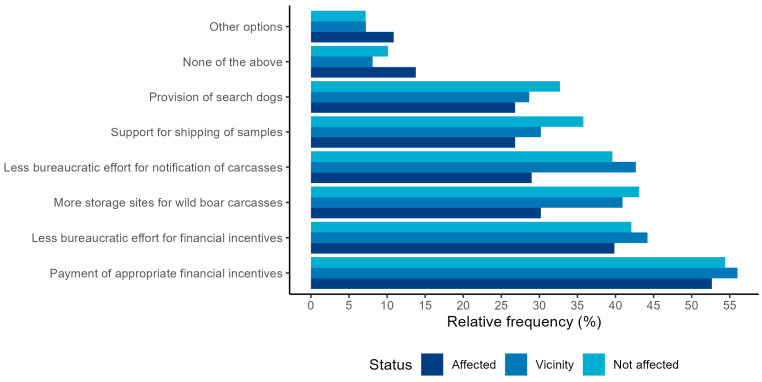
Relative frequency (in %) of hunters from ASF-affected areas (*n* = 414), hunters from areas in the vicinity of ASF (*n* = 457), and hunters from non-affected federal states (*n* = 682) who selected the respective options in a web-based questionnaire for German hunters as motivation to increase their participation in the search for and sampling of wild boar carcasses in terms of ASF surveillance and control.

The top three motivational options were the payment of appropriate financial incentives (a: 52.7%, b: 56.0%, c: 54.4%), the reduction in bureaucratic effort for receiving financial incentives (a: 39.9%%, b: 44.2%, c: 42.1%), and the increase in the number of collection and storage sites for wild boar carcasses (a: 30.2%, b: 40.9%, c: 43.1%) (Figure 7). The proportion of hunters who selected an increased number of storage sites for carcasses and the reduction in bureaucracy for the notification of wild boar (a: 42.7%, b: 39.6%, c: 29.0%) was significantly higher in the group of hunters in the vicinity (*p* = 0.001, *p* < 0.001) and hunters from non-affected regions (*p* < 0.001) compared to hunters from ASF-affected areas. Moreover, a significantly larger proportion of hunters from unaffected regions selected support for shipping of samples (c: 35.8%, a: 26.8%) as a motivational option compared to hunters from ASF-affected areas (*p* = 0.003). No statistically significant differences were detected between the choices of hunters in the vicinity and non-affected hunters (Supplementary Table S20).

A total of 127 participants suggested other options that could motivate them to increase their participation in the search for wild boar carcasses in the free text (Supplementary Table S21). The most frequently mentioned option (20.5%) was the payment of financial compensation for participation based on the time invested rather than the number of detected carcasses. Improving cooperation with the veterinary authorities, in particular, the flow of information and coordination of control measures, was also seen as a motivating factor by 12.6%. In addition, 10.2% considered increased opportunities or support to train their dog to search for carcasses and the possibility of taking paid time off work to participate in carcass searches as motivating.

### 3.6. Hunter’s Additional Comments

A total of 294 participants left some additional comments before submitting the questionnaire (Supplementary Table S22). Of these, 7.5% stated that they had nothing further to say, and 10.2% made positive comments about the questionnaire, such as that they were happy to support the work or were grateful for the opportunity to express their opinions. In contrast, 3.7% were critical of the questionnaire. In addition, a large number of aspects mentioned in previous free-text responses were repeated. The most common (10.2%) was the criticism of fencing due to its negative impact on other wildlife species and the fragmentation of hunting areas. Furthermore, 7.1% expressed the wish to improve cooperation and communication with the authorities and 6.1% stressed the importance of raising awareness among the general public and stakeholders about ASF and its surveillance and control.

## 4. Discussion

ASF has been present in Germany since September 2020 and it mainly affects the wild boar population [10]. The circulation of ASF in wild boar populations poses a constant risk of spreading to pig farms, which can lead to major socio-economic losses and negative impacts on animal welfare [13]. Hunters are key players in implementing measures for ASF surveillance and control of wild boar, including carcass searches, the sampling of wild boar, and increased hunting to reduce the susceptible wild boar population. 

A questionnaire study distributed among the German hunting community was conducted to elucidate which tasks hunters perform in ASF surveillance and control and how they perceive the effectiveness of these tasks, which obstacles they face when participating in ASF surveillance and control, and which options they consider to be motivating to increase their participation in certain activities. 

As this was a public web-based survey, it cannot be ruled out that people participated who were not hunters. To address this issue, the survey mainly consisted of mandatory questions, and only fully completed questionnaires were analyzed. Answering the mandatory questions required a deep knowledge of hunting practices. This made it unlikely that a substantial number of people who were not hunters completed the questionnaire. Moreover, the survey link was made available through organizations associated with hunting, which made it less likely that members of the general public had access. Also, no outlying responses became apparent when managing and analyzing data. It can, therefore, be assumed that the vast majority of the participants were hunters engaged or interested in the topic of ASF prevention, surveillance, and control or otherwise stopped answering when confronted with the first part of the questionnaire. This view is also supported by the analysis of the dropout ratios per page. In addition, a larger number of participants dropped out when asked about which measures of ASF surveillance and control they had participated in, probably because the question was too complex, the instructions on how to answer it were unclear, or the participants had not been involved in such measures but were reluctant to admit this. 

In total, 1553 participants completed the questionnaire, which represents a small group of approximately 403,000 hunters in Germany [33]. However, the repetition of codes throughout the free-text answers to a point where almost no new codes were identified in the data, suggesting that inductive thematic saturation may have already been reached [34,35]. 

The demographics of the participants in this study were similar to the results of a study of the hunting community by the German Hunting Association [33]. Nevertheless, a possible gender and age bias in the results of our study has to be taken into consideration. 

Unsurprisingly, the level of participation in the questionnaire was particularly high in the ASF-affected federal states. However, responses from all federal states were received, showing that hunters outside affected areas are highly interested in ASF. Overall, few significant differences were found between the responses of hunters in the vicinity of ASF and non-affected hunters, suggesting that hunters’ perceptions are not strongly influenced by the proximity of the epidemic front as long as their own hunting area is not affected. 

In contrast to the present study, where less than a third of the hunters believed that ASF could be successfully eliminated in wild boar in Germany, the proportion (70%) was considerably higher in Lithuania [26]. This difference in the attitude of the hunters may be influenced by the epidemiological situation in the countries at the time of the questionnaire. This highlights the necessity to address hunters’ perceptions and concerns toward ASF control to keep up their motivation to participate in surveillance and control measures in the long term.

Similar to their Lithuanian colleagues, German hunters mainly rated their knowledge of ASF as good or very good [26]. Therefore, affected hunters rated their knowledge of ASF significantly better than hunters in the vicinity of ASF and non-affected hunters, probably because of more experience and personal exposure to the disease. However, the self-evaluation of knowledge was not validated in the questionnaire. The listed sources of information indicate that hunters are generally interested in learning more about ASF, and media specifically created for hunters should be used primarily to provide hunters with relevant information and to increase awareness.

Overall, German hunters were rather satisfied with the cooperation with other stakeholders or remained neutral. Differences in hunters’ satisfaction with hunting associations in different regions may be due to regional differences in the importance and structure of these associations, irrespective of the ASF status of the region. In the affected federal state of Saxony, the proportion of hunters who are members of these associations is considerably smaller than in other federal states [36]. The results indicate that it is only after an ASF outbreak that cooperation between the different actors at the local level is intensified, and this local cooperation mainly works satisfactorily in the event of an outbreak. However, conflicts with other hunters or other stakeholders were mentioned as a consequence of ASF control and surveillance, illustrating the big challenge of implementing ASF prevention, surveillance, and control measures [37]. Likewise, in a participatory study conducted by Jori et al., experts judged that ASF control has an impact on a large panel of stakeholders and concluded that “communication among and between stakeholders seems to be both difficult and essential”. Yet, it is necessary to involve these stakeholders [38]. The desire for improved communication was expressed by participants in this study in their free-text answers and also by hunters from the Baltic states in previous studies [24,25]. This highlights the need for transparent and rapid communication and the need to prepare communication channels at an early stage in order to respond effectively to new outbreaks. 

Overall, German hunters seem to assess the effectiveness of measures that support hunting to be more effective and rate hunting bans as the least effective measures of ASF control, which is in accordance with the results of other studies [23,26,27] but may also show some level of “vested interest”. 

The results may indicate that hunters in the vicinity of ASF and those from areas not affected by ASF are more optimistic about the success of ASF control than hunters from affected areas. They might have resigned due to the fact that the disease is still present after several months of ASF control, although great efforts were made and apparently led to an increase in personal workload and financial expenses. This burden was also reported by hunters from the Baltic states [23,27]. German hunters apparently also experienced an increase or reduction in their hunting activities, depending on the area. In affected regions, hunting activity was more likely to decrease due to hunting bans and a reduced wild boar population. By contrast, the consequence can also be an increase in hunting activity due to increased hunting as a surveillance measure in regions that have yet to be affected. Hunters have to face the moral challenge of a substantial reduction in the wild boar population in their hunting area and the need to deal with local restrictions on their hunting activity due to the establishment of restriction zones in affected areas or fencing. It was reported by Stončiūtė et al. that some hunters lost their joy and motivation for hunting [27]. 

Although the search for carcasses was rated as fairly effective by the hunting community, a substantial proportion of the participants (10%) did not find any motivation to increase their participation in this measure. This is understandable since the search for wild boar carcasses can be a time-consuming and sometimes tedious activity. It is also rated as less practical by some experts [19]. Likewise, Lithuanian hunters considered going into the forest specifically to search for wild boar carcasses to be less effective and were less willing to support this measure in comparison to searching for carcasses while they were already out in the forest [26]. However, several studies have shown that the search for carcasses is of utmost importance in the surveillance and control of ASF in wild boar since carcasses left in the forest pose a risk of infection to living wild boar and carcass sampling is useful for detecting new introductions of ASF [11,16,19]. 

Similar to hunters from the Baltic states [24,25,26], German hunters considered a payment of appropriate financial compensation and a reduction in bureaucracy as motivational options to increase participation in carcass search and wild boar hunting. The exact amount of money was not defined in the questionnaire since there are regionally different regulations in the German districts and federal states regarding eligibility and sums. For example, the compensation for hunted wild boar ranged from EUR 70 per animal in the non-affected federal state of Bavaria (Bayerisches Landesamt für Gesundheit und Lebensmittelsicherheit, https://www.lgl.bayern.de/tiergesundheit/tierkrankheiten/virusinfektionen/asp/infos_jaeger.htm#aufwand, accessed on 28 August 2023) to EUR 150 per animal in the affected federal state of Brandenburg (Ministerium für Soziales, Gesundheit, Integration und Verbraucherschutz des Landes Brandenburg), https://msgiv.brandenburg.de/msgiv/de/themen/verbraucherschutz/veterinaerwesen/tierseuchen/afrikanische-schweinepest/, accessed on 28 August 2023). In our study, a significantly larger proportion of affected hunters stated to receive payments, which might have contributed to the significantly greater hunting bag and greater hunting frequency of these hunters. However, approximately one-fifth of the participants stated that they had not received any financial rewards. This could be due to the fact that there are no rewards in the participants’ region or that not every hunter is eligible to receive the payments. Therefore, depending on the individual situation, the understanding of the “appropriate” amount may differ and could mean either increasing rewards or introducing rewards in general. In areas not affected by ASF, a predominant lack of available storage sites for carcasses and sample drop-off points might be an obstacle for carcass search and sampling. This stresses the need for good preparation and the establishment of infrastructure for the implementation of control measures at an early stage. In affected areas, the possibility of making use of and selling (ASF-negative) wild boar meat and products appeared to be of greater concern for hunters. Although increased hunting to reduce the susceptible wild boar population as a measure to hinder the spread of ASF was also assessed as a fairly effective measure, this strategy was perceived controversially by participants. Likewise, Oelke et al. reported different opinions on the topic that were gathered in interviews with German hunters [37]. Some hunters considered increased hunting as an unnecessary culling of wild animals to protect the domestic pig industry, opposing their ethical framework of fair hunting *(“Waidgerechtigkeit”)* [37,39]. In addition, restrictions in affected areas on selling and distributing wild boar meat as well as value loss of the meat are in contrast to the ethical standards and traditions of hunters to make use of the products of hunted animals. These issues may lead to reduced acceptance and compliance among hunters and should be addressed in order to keep up hunters’ motivation and participation. However, it is important to note and communicate that the increased hunting of wild boar in the context of ASF control cannot be described as leisure hunting [40] but rather as a necessary component of ASF control.

Similar to German hunters, Lithuanian hunters considered the permission to use additional hunting tools as an effective measure to eliminate ASF [26]. As nighttime hunting can be challenging, the use of such aids may contribute to more effective hunting. However, some participants considered their use to be unethical as they stand against the traditional perception of fair hunting. This controversy was also reported by Oelke et al. [37].

Likewise, the construction of fences and the use of wild boar traps to reduce the wild boar population appear to be a controversial issue among German hunters. The construction of fences can interfere with individual property rights and has a strong impact on ecosystems of wildlife, eventually leading to lower acceptance of this measure [38]. Estonian and Latvian hunters also found fencing to be ineffective and a waste of time and money [24,25]. Even among other experts, there is disagreement on the effectiveness of fencing in controlling ASF [38], which seems to be highly dependent on the local situation of the outbreak. However, it was used successfully to control the focal ASF outbreak in the Czech Republic [41] and Belgium and has contributed to controlling or at least slowing down the westward spread of ASF in Germany [42]. 

Capturing and culling wild boar in traps was considered by experts as a feasible complementary measure to reduce the wild boar population in the event of an ASF outbreak, allowing for high biosecurity standards, but was opposed by hunters [38]. Similarly, the results of this study suggest that there is a disagreement among German hunters on this issue. Moreover, the assessment of the effectiveness of selective hunting of adult female wild boar was also heterogeneous. This measure was considered an unethical hunting practice that increases the risk of producing orphans [23,27]. 

Similar to hunters from Latvia and Lithuania [25,26], increased biosecurity, e.g., the cleaning and disinfection of hunting equipment, clothing, and vehicles was considered to be fairly effective by German hunters. It is vital that the hunting community is aware of the importance of increased biosecurity measures and is educated on how to implement them, as a notable proportion of German hunters reported that they travel to other European countries for hunting and have regular contact with domestic pig farms, which poses a risk for transmission of ASF through contaminated fomites.

## 5. Conclusions

Overall, the general perception of the effectiveness of specific ASF surveillance and control measures is consistent with the results of other studies in different countries. Consistently, measures that promote hunting were considered effective, whereas measures that hindered hunting were considered ineffective. The consequences for hunters were also perceived in a similar way, mainly in terms of an increase in workload and financial expenses. Therefore, financial compensation and a reduction in bureaucracy were consistently considered as motivational options. Intervention studies would be required to evaluate the real impact of these motivators. By showing regional differences between hunters affected by ASF, hunters in the vicinity of ASF, and non-affected hunters, our study highlights the necessity to consider hunters’ perceptions and opinions on ASF surveillance and control to maintain their participation and motivation in the long term. The establishment of ways of communication between and among stakeholders is of utmost importance and must be in place early in preparation for potential outbreaks to ensure consistent education and flow of information about the ASF situation. It is of utmost importance to consider local hunters’ perceptions and address their concerns at an early stage to increase their compliance when it comes to implementing restrictive control measures. This study highlights various challenges of bringing together different stakeholders, such as hunters, farmers, or authorities, in the context of ASF control and may thus indicate the need for inter-sectoral and complex approaches involving different stakeholders to identify adaptive solutions.

## Figures and Tables

**Table 1:** Demographic information of the participants (*n* = 1553). The table shows the relative proportion (in %) of gender, age, and years of hunting experience of the participants in a web-based questionnaire study for German hunters.

Gender (%)		Age (%)		Hunting Experience (%)	
Female	12.1	Under 20 Years	1.5	Under 3 Years	5.7
Male	87.4	20 to 40 years	27.9	3 to 10 years	25.7
Diverse	0.5	41 to 60 years	47.0	11 to 30 years	40.2
		Over 60 years	23.6	Over 30 years	28.4

**Table 2:** Proportion (in %) of hunters from ASF-affected regions (*n* = 414) in the vicinity of ASF (*n* = 457) and non-affected regions (*n* = 682) that hunted less than 1 time, 1 to 5 times, 6 to 10 times or more than 10 times per month as mentioned in a web-based questionnaire study for German hunters. Hunters from affected areas and in the vicinity of ASF hunted significantly more often than hunters from non-affected areas (*p* < 0.001 and *p* = 0.014). No statistically significant difference was detected between affected areas and vicinity areas (*p* = 0.074).

	Affected (%)	Vicinity (%)	Not Affected (%)
Less than 1 time	4.8	4.8	8.9
1 to 5 times	26.8	33.3	37.1
6 to 10 times	33.1	34.1	29.6
More than 10 times	35.3	27.8	24.3

**Table 3:** The median level of effectiveness of respective ASF control and surveillance measures as assessed by hunters from ASF-affected areas (*n* = 414), hunters in the vicinity of ASF (*n* = 457), and hunters from non-affected federal states (*n* = 682) in a web-based questionnaire for German hunters with the following levels: very effective (1), fairly effective (2), moderately effective (3), hardly effective (4), or not effective (5). Relative frequency (in %) of hunters who selected the alternative option (“I don’t know”) rather than assessing the effectiveness of respective measures. For the analysis of the median, the proportion of hunters who selected the alternative option (“I don’t know”) was excluded (“Excluded”).

	Affected		Vicinity		Not Affected	
	Median	Excluded (%)	Median	Excluded (%)	Median	Excluded (%)
Sampling of wild boar (hunted or found dead)	2	1.2	2	3.9	2	2.6
Increased hunting of wild boar	2	0.2	2	2.0	2	1.8
Increased hunting of young animals	2	1.0	2	2.8	2	2.6
Intensive search for and removal of wild boar carcasses	2	0.7	2	3.3	2	3.4
Cleaning + disinfection of hunting equipment, clothing, and vehicles	2	3.6	2	5.7	2	4.4
Increased hunting of adult females	3	3.4	2	3.9	2	4.8
Use of wild boar traps	3	13.3	3	16.4	3	19.9
Construction of fences	3	3.4	3	8.5	3	9.5
Temporary ban on driven hunts after ASF outbreak	4	2.7	3	7.0	3	6.3
Temporary ban on hide hunting after ASF outbreak	5	2.4	4	8.5	4	9.4
Permanent ban on driven hunts after ASF outbreak	5	1.4	4	9.0	4	7.6
Temporary ban on any hunting activity after ASF outbreak	5	1.4	4	6.3	4	5.7
Permanent ban on hide hunting after ASF outbreak	5	1.7	5	7.2	5	7.9
Permanent ban on any hunting activity after ASF outbreak	5	1.2	5	5.9	5	5.3

**Table 4:** Relative frequency (in %) of hunters who stated to expect or experience the respective consequences of ASF surveillance and control for hunters from ASF-affected areas (*n* = 414), hunters in the vicinity of ASF (*n* = 457), and hunters from regions not affected by ASF (*n* = 682) as mentioned in a web-based questionnaire for German hunters.

	Affected (%)	Vicinity (%)	Not Affected (%)
Reduction in the wild boar population in the hunting area	78.0	71.6	69.6
Increased personal work and time load	73.4	73.7	78.9
Increased personal costs	60.9	53.8	55.0
Local restrictions of own hunting activity	54.1	47.5	51.3
Increase in own hunting activity (single hunt)	40.6	56.7	58.2
Reduction in own hunting activity (single hunt)	38.6	22.1	22.6
Conflicts with other hunters	20.5	22.5	19.1
Conflicts with farmers	17.6	23.0	32.1
Conflicts with veterinary authorities	21.5	19.9	21.4
Other consequences	14.0	7.7	8.1
No consequences	1.7	3.3	4.3

**Table 5:** Assessment of the satisfaction with cooperation and appreciation by other stakeholders of hunters from ASF-affected regions (*n* = 414) as mentioned in a web-based questionnaire for German hunters. The table shows the median level of satisfaction of hunters’ cooperation with respective stakeholders and the level of appreciation of hunters and their work by respective stakeholders. Levels 2 and 3 correspond to the answers “Rather satisfied” and “Neutral” regarding the level of satisfaction and “Rather yes” and “Neutral” regarding the question of whether they felt valued. To calculate the median, the share of hunters that selected the answer options “No cooperation” (no coop.) and “No specification” (no spec.) was excluded. The proportion (in %) of hunters that selected alternative answer options out of the total responses of hunters from affected areas (*n* = 414) is displayed.

	Satisfaction			Appreciation		
	Median	No Coop. (%)	No Spec. (%)	Median	No Coop. (%)	No Spec. (%)
Local veterinary authority	2	2.7	0.7	2	1.7	3.6
Hunting ring	2	6.5	2.4	2	5.3	5.1
State laboratory	3	11.6	8.7	3	9.9	11.8
External forces	3	21.0	6.8	3	18.6	10.1
Local agriculture	3	10.4	2.7	3	6.3	5.3
State hunting association	3	13.0	3.1	3	10.9	6.8
National hunting association	3	21.5	5.8	3	17.1	9.2

**Table 6:** Assessment of the satisfaction with cooperation and appreciation by other stakeholders of hunters from regions in the vicinity of ASF occurrence (*n* = 457) as mentioned in a web-based questionnaire for German hunters. The table shows the median level of satisfaction of hunters’ cooperation with respective stakeholders and the level of appreciation of hunters and their work by respective stakeholders. Levels 2 and 3 correspond to the answers “Rather satisfied” and “Neutral” regarding the level of satisfaction and “Rather yes” and “Neutral” regarding the question of whether they felt valued. To calculate the median, the share of hunters that selected the answer options “No cooperation” (no coop.) and “No specification” (no spec.) was excluded. The proportion (in %) of hunters that selected alternative answer options out of the total responses of hunters from areas in the vicinity of ASF occurrence (*n* = 457) is displayed.

	Satisfaction			Appreciation		
	Median	No Coop. (%)	No Spec. (%)	Median	No Coop. (%)	No Spec. (%)
Local veterinary authority	2	5.0	4.2	3	5.0	8.3
Hunting ring	2	7.2	5.5	2	4.6	7.9
State laboratory	3	17.3	10.5	3	14.9	13.1
External forces	3	23.6	12.5	3	20.1	16.6
Local agriculture	3	10.9	7.4	3	7.9	8.3
State hunting association	3	10.5	4.4	3	7.9	7.4
National hunting association	3	15.8	7.9	3	12.7	9.2

**Table 7:** Assessment of the satisfaction with cooperation and appreciation by other stakeholders of hunters from regions not affected by ASF (*n* = 682) as mentioned in a web-based questionnaire for German hunters. The table shows the median level of satisfaction of hunters’ cooperation with respective stakeholders and the level of appreciation of hunters and their work by respective stakeholders. The median levels 2 and 3 correspond to the answers “Rather satisfied” and “Neutral” regarding the level of satisfaction and “Rather yes” and “Neutral” regarding the question of whether they felt valued. To calculate the median, the share of hunters that selected the answer options “No cooperation” (no coop.) and “No specification” (no spec.) was excluded. The proportion (in %) of hunters that selected alternative answer options out of the total responses of hunters from non-affected areas (*n* = 682) is displayed.

	Satisfaction			Appreciation		
	Median	No Coop. (%)	No Spec. (%)	Median	No Coop. (%)	No Spec. (%)
Local veterinary authority	3	8.9	5.9	3	7.9	6.5
Hunting ring	2	5.9	5.0	2	5.0	5.7
State laboratory	3	22.1	10.6	3	19.2	10.6
External forces	3	26.7	15.4	3	24.6	14.4
Local agriculture	3	13.3	7.0	3	9.2	6.0
State hunting association	2	7.8	4.1	2	6.0	5.4
National hunting association	3	12.8	6.3	3	9.7	7.3

## Data Availability

The anonymized data used for the analysis can be obtained from the corresponding author upon reasonable request.

## Supplementary Materials

The following supporting information can be downloaded at https://www.mdpi.com/article/10.3390/ani13182813/s1, Document S1: Original questionnaire in the German language; Document S2: English translation of the questionnaire; Supplementary Table S1. Analysis of the free-text answers for different options of hunting organization; Supplementary Table S2. Analysis of the given free-text reasons why hunters believe that the elimination of ASF in wild boar in Germany is possible; Supplementary Table S3. Analysis of the given free-text reasons why hunters believe that the elimination of ASF in wild boar in Germany is not possible; Supplementary Table S4. Analysis of the given free-text reasons why hunters were unsure whether the elimination of ASF in wild boar in Germany is possible or not; Supplementary Table S5. Analysis of the given free-text reasons why hunters believe that they play a crucial role in ASF control in Germany; Supplementary Table S6. Analysis of the given free-text reasons why hunters believe that they do not play a crucial role in ASF control in Germany; Supplementary Table S7. Analysis of the given free-text reasons why hunters were unsure whether they play a crucial role in ASF control in Germany or not; Supplementary Table S8. Analysis of the free-text answers for further sources used to obtain knowledge about ASF; Supplementary Table S9. Analysis of the free-text answers for further measures that were implemented by hunters in terms of ASF surveillance or control; Supplementary Table S10. Pairwise Mann–Whitney U testing (with the Bonferroni correction) of the assessment of the effectiveness of different ASF-surveillance and control; Supplementary Table S11. Analysis of the free-text answers for additional effective measures to control ASF; Supplementary Table S12. Chi-squared testing of the selected consequences of ASF surveillance and control, which hunters from ASF-affected areas, hunters in the vicinity of ASF, and hunters from non-affected areas expect or experience; Supplementary Table S13. Analysis of the free-text answers for additional consequences of ASF surveillance and control; Supplementary Table S14. Pairwise Mann–Whitney U testing (with the Bonferroni correction) of the assessment of hunters’ satisfaction with cooperation with other stakeholders involved in ASF surveillance and control; Supplementary Table S15. Pairwise Mann–Whitney U testing (with the Bonferroni correction) of the assessment of whether the hunters felt appreciated by other stakeholders involved in ASF surveillance and control; Supplementary Table S16. Analysis of the given free-text reasons why none of the provided options were considered to be motivational to increase participation in wild boar hunting; Supplementary Table S17. Chi-squared testing of the selected motivational options that would likely increase hunters’ participation in the increased hunting of wild boar in terms of ASF surveillance and control; Supplementary Table S18. Analysis of the free-text answers for additional motivational options to increase participation in hunting wild boar; Supplementary Table S19. Analysis of the given free-text reasons why none of the provided options were considered to be motivational to increase participation in carcass search; Supplementary Table S20. Chi-squared testing of the selected motivational options that would likely increase hunters’ participation in the search for wild boar carcasses in terms of ASF surveillance and control; Supplementary Table S21. Analysis of the free-text answers for additional motivational options to increase participation in carcass searches; Supplementary Table S22. Analysis of the additional free-text remarks or comments in the questionnaire.
