# Supplementary material for: African Swine Fever in Wild Boar: German Hunters’ Perception of Surveillance and Control—A Questionnaire Study
Source: Animals (Basel). 2023 Sep 5;13(18):2813. doi: 10.3390/ani13182813 (PMC10525383; doi:10.3390/ani13182813)
Supplement: Supplementary file 1 [file animals-13-02813-s001.zip › Supplementary_Document_S1.pdf]

fragebogen-asp → base 23.05.2022, 11:41

Seite 01

Bekämpfung der Afrikanischen Schweinepest in Deutschland

Die Afrikanische Schweinepest (ASP) hat Deutschland im September 2020 erreicht. Trotz intensiver Bemühungen und dem Einsatz umfassender Bekämpfungsmaßnahmen, breitet sie sich weiter in der deutschen Wildschweinpopulation aus.

In der Bekämpfung der ASP beim Wildschwein spielen lokale Einsatzkräfte, wie Jäger*innen, eine entscheidende Rolle: Sie kennen die Gegebenheiten vor Ort besonders gut und haben langjährige Erfahrung im Umgang mit Schwarzwild. Aus diesem Grund möchten wir mit diesem Fragebogen einen Einblick in Ihre Meinungen, Ideen und Bedenken als Jäger*innen bezüglich der Unterstützung von Bekämpfungsmaßnahmen gegen die ASP beim Wildschwein erhalten. Mit den Ergebnissen möchten wir die bestehenden Strategien hinsichtlich ihrer Umsetzbarkeit bewerten und Empfehlungen für Anpassungen erarbeiten.

Die Beantwortung des Fragebogens wird ungefähr 15 Minuten in Anspruch nehmen. Bitten nehmen Sie nur an der Umfrage teil, wenn Sie tatsächlich aktive*r Jäger*in sind. Hinweis: Die aufgeführten Fragen beziehen sich alle ausschließlich auf die ASP beim Wildschwein.

Mit dem Ausfüllen dieses Fragebogens erklären Sie sich damit einverstanden, dass die gewonnenen Daten in anonymisierter Form für Forschungszwecke und Veröffentlichungen verwendet werden.

Herzlichen Dank für Ihre Teilnahme!

Seite 02

1. Stimmen Sie der folgenden Aussage zu?

„Die Eliminierung der ASP in der deutschen Wildschweinpopulation ist möglich.“

○ Ich stimme zu. ○ Ich stimme nicht zu. ○ Ich weiß es nicht.

Warum stimmen Sie zu bzw. stimmen Sie nicht zu? Bitte begründen Sie Ihre Entscheidung.

2. Stimmen Sie der folgenden Aussage zu?

„Jäger*innen spielen in der Bekämpfung der ASP in Deutschland eine ENTSCHEIDENDE Rolle.“

○ Ich stimme zu. ○ Ich stimme nicht zu. ○ Ich weiß es nicht.

Warum stimmen Sie zu bzw. stimmen Sie nicht zu? Bitte begründen Sie Ihre Entscheidung.

Seite 03

3. Wie schätzen Sie Ihr Wissen über die ASP ein?

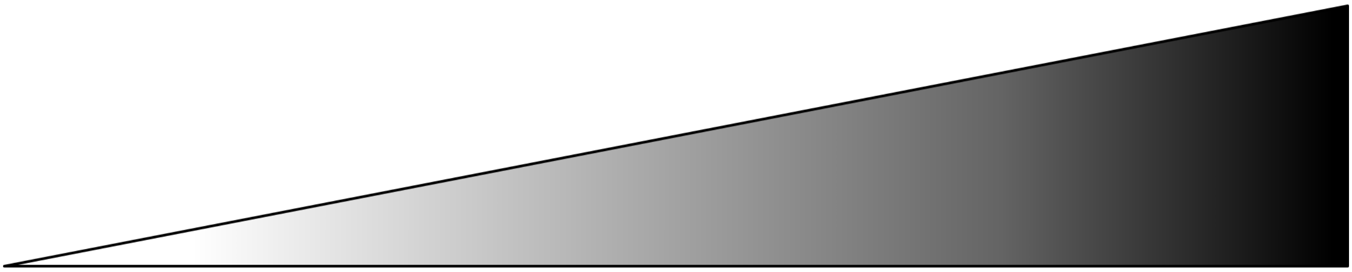

○ Sehr schlecht ○ Eher schlecht ○ Mäßig ○ Eher gut ○ Sehr gut

4. Woher und auf welchem Weg beziehen Sie ihr Wissen bzw. Ihre Informationen über die ASP?

Eine Mehrfachauswahl ist möglich.

- ☐ Zeitungen und Zeitschriften für Jäger*innen
- ☐ Schriftliche Informationen des Jagdverbandes
- ☐ Schriftliche Informationen des lokalen Veterinäramts
- ☐ Schriftliche Informationen der Forschungs- und Untersuchungseinrichtungen (Landeslabore, Friedrich-Loeffler-Institut)
- ☐ Websites bzw. Internetforen für Jäger*innen
- ☐ Website des Jagdverbandes
- ☐ Website des lokalen Veterinäramts
- ☐ Website der Forschungs- und Untersuchungseinrichtungen (Landeslabore, Friedrich-Loeffler-Institut)
- ☐ Soziale Medien (Facebook, Instagram, Twitter)
- ☐ Fernsehen (Nachrichten, Dokumentationen)
- ☐ Persönliche Gespräche mit anderen Jäger*innen
- ☐ Persönliche Gespräche mit dem lokalen Veterinäramt
- ☐ Veranstaltungen (Messen, Vorträge)
- ☐ Weitere Quellen: Bitte teilen Sie uns mit, aus welchen weiteren Quellen Sie ihr Wissen beziehen.

- ☐ Keine Angabe.

Seite 04

5. Wie zufrieden sind Sie mit der Zusammenarbeit mit den jeweiligen genannten Einrichtungen und Personengruppen in Hinblick auf die ASP-Bekämpfung und Vorbeugung in den letzten 12 Monaten?

	Sehr zufrieden	Eher zufrieden	Neutral	Eher unzufrieden	Sehr unzufrieden	Keine Zusammen-arbeit	Keine Angabe
Örtliche Jägerschaft / Hegering	○	○	○	○	○	○	○
Landesjagdverband	○	○	○	○	○	○	○
Bundesjagdverband	○	○	○	○	○	○	○
Örtliche Landwirtschaft	○	○	○	○	○	○	○
Veterinäramt des Landkreises	○	○	○	○	○	○	○
Landeslabor	○	○	○	○	○	○	○
Externe Einsatzkräfte (z.B. THW, Bundeswehr, freiwillige Helfer für Kadaversuche/Zaunbau)	○	○	○	○	○	○	○

6. Fühlen Sie sich und Ihre Arbeit durch die jeweils genannten Einrichtungen und Personengruppen angemessen wertgeschätzt?

	Ja	Eher ja	Neutral	Eher nein	Nein	Keine Zusammen-arbeit	Keine Angabe
Örtliche Jägerschaft / Hegering	○	○	○	○	○	○	○
Landesjagdverband	○	○	○	○	○	○	○
Bundesjagdverband	○	○	○	○	○	○	○
Örtliche Landwirtschaft	○	○	○	○	○	○	○
Veterinäramt des Landkreises	○	○	○	○	○	○	○
Landeslabor	○	○	○	○	○	○	○
Externe Einsatzkräfte (z.B. THW, Bundeswehr, freiwillige Helfer für Kadaversuche/Zaunbau)	○	○	○	○	○	○	○

Seite 05

7. Welche Auswirkungen erleben oder erwarten Sie durch die Maßnahmen zur ASP-Vorbeugung und Bekämpfung?

Eine Mehrfachauswahl ist möglich.

- ☐ Verringerung der eigenen Jagdaktivität (Einzeljagd)
- ☐ Erhöhung der eigenen Jagdaktivität (Einzeljagd)
- ☐ Örtliche Einschränkungen der eigenen Jagdaktvität
- ☐ Verkleinerung der Wildschweinpopulation im Jagdgebiet
- ☐ Erhöhter persönlicher Arbeits- und Zeitaufwand
- ☐ Erhöhte persönliche Kosten
- ☐ Konflikte mit Veterinärämtern
- ☐ Konflikte mit anderen Jäger*innen
- ☐ Konflikte mit Landwirt*innen
- ☐ Weitere Auswirkungen
  Bitte teilen Sie uns mit, welche weiteren Auswirkungen Sie erleben bzw. erwarten.

- ☐ Ich erlebe bzw. erwarte keine Auswirkungen durch Maßnahmen zur ASP-Vorbeugung und Bekämpfung.

8. An welchen Maßnahmen zur Vorbeugung oder Bekämpfung der ASP haben Sie in den letzten 12 Monaten teilgenommen?

Eine Mehrfachauswahl ist möglich.

Beprobung von auffällig erlegten bzw. tot gefunden Wildschweinen

Zur Vorbeugung in nicht betroffenen Gebieten | Zur Tierseuchenbekämpfung in betroffenen Gebieten | *Nicht durchgeführt*

Fallwildsuche im Rahmen des üblichen Waldgangs

Zur Vorbeugung in nicht betroffenen Gebieten | Zur Tierseuchenbekämpfung in betroffenen Gebieten | *Nicht durchgeführt*

Organisierte Fallwildsuche (z.B. mit Menschenketten, Hunden, Drohnen)

Zur Vorbeugung in nicht betroffenen Gebieten | Zur Tierseuchenbekämpfung in betroffenen Gebieten | *Nicht durchgeführt*

Beseitigung von Fallwild

Zur Vorbeugung in nicht betroffenen Gebieten | Zur Tierseuchenbekämpfung in betroffenen Gebieten | *Nicht durchgeführt*

Vermehrte Bejagung von Schwarzwild

Zur Vorbeugung in nicht betroffenen Gebieten | Zur Tierseuchenbekämpfung in betroffenen Gebieten | *Nicht durchgeführt*

Vermehrte Jagd erwachsener weiblicher Tiere

Zur Vorbeugung in nicht betroffenen Gebieten | Zur Tierseuchenbekämpfung in betroffenen Gebieten | *Nicht durchgeführt*

Einsatz von Großfallen

Zur Vorbeugung in nicht betroffenen Gebieten | Zur Tierseuchenbekämpfung in betroffenen Gebieten | *Nicht durchgeführt*

Errichtung von Zäunen

Zur Vorbeugung in nicht betroffenen Gebieten | Zur Tierseuchenbekämpfung in betroffenen Gebieten | *Nicht durchgeführt*

9. Gibt es weitere Maßnahmen zur Vorbeugung bzw. Bekämpfung der ASP, an denen Sie teilgenommen haben? Wenn ja, teilen Sie uns diese bitte hier mit.

10. Wie bewerten Sie die Effektivität der genannten Maßnahmen in Hinblick auf eine erfolgreiche Bekämpfung der ASP?

	Sehr effektiv	Ziemlich effektiv	Mäßig effektiv	Wenig effektiv	Nicht effektiv	Ich weiß nicht.
Beprobung von auffällig/krank erlegten bzw. tot gefunden Wildschweinen	○	○	○	○	○	○
Intensive Suche nach und Beseitigung von Fallwild	○	○	○	○	○	○
Vermehrte Bejagung von Schwarzwild	○	○	○	○	○	○
Vermehrte Jagd auf erwachsene, weibliche Tiere	○	○	○	○	○	○
Vermehrte Jagd auf Jungtiere	○	○	○	○	○	○
Einsatz von Großfallen	○	○	○	○	○	○
Errichtung von Zäunen	○	○	○	○	○	○
Reinigung + Desinfektion von Jagdutensilien/-bekleidung und Fahrzeugen	○	○	○	○	○	○
Vorrübergehendes Verbot von Ansitzjagden nach ASP-Ausbruch	○	○	○	○	○	○
Dauerhaftes Verbot von Ansitzjagden in betroffenen Gebieten	○	○	○	○	○	○
Vorrübergehendes Verbot von Treib- oder Drückjagden nach ASP-Ausbruch	○	○	○	○	○	○
Dauerhaftes Verbot von Treib- oder Drückjagden in betroffenen Gebieten	○	○	○	○	○	○
Vorrübergehendes Verbot von jeglicher Jagdaktivität nach ASP-Ausbruch	○	○	○	○	○	○
Dauerhaftes Verbot von jeglicher Jagdaktivität in betroffenen Gebieten	○	○	○	○	○	○

11. Gibt es weitere Maßnahmen, die hier nicht erwähnt sind, die Sie als sehr effektiv einstufen?

Bitte teilen Sie uns diese hier mit.

Seite 08

12. Wie häufig gehen Sie durchschnittlich pro Monat zur Jagd auf Wildschweine?

- ◯ Weniger als 1 Mal
- ◯ 1 bis 5 Mal
- ◯ 6 bis 10 Mal
- ◯ Über 10 Mal

13. Nutzen Sie bereits Schalldämpfer oder Nachtsichttechnik für die Jagd auf Wildschweine?

- ◯ Nachtsichttechnik
- ◯ Schalldämpfer
- ◯ Beides
- ◯ Keines von beiden

14. Wären Sie im Falle eines Seuchenausbruchs der ASP bereit, Großfallen zu bedienen und Schwarzwild in diesen Fallen zu eliminieren?

◯ Ja ◯ Nein

15. Welche Prämien oder Aufwandsentschädigungen im Rahmen der ASP-Vorbeugung und Bekämpfung können Sie in Ihrem Bundesland einfordern?

- ☐ Prämien für erlegtes Schwarzwild
- ☐ Prämien für die Meldung von Fall- bzw. Unfallwild
- ☐ Prämien für die Beprobung von Schwarzwild

- ☐ Ich weiß es nicht.
- ☐ Keine.

Seite 09

16. Welche der genannten Optionen würde Sie mit hoher Wahrscheinlichkeit dazu motivieren, zur Verkleinerung der Schwarzwildpopulation häufiger zur Jagd zu gehen bzw. vermehrt Schwarzwild zu erlegen?

- ☐ Bereitstellung von zusätzlichen Hilfsmitteln, wie z.B. Nachtsichttechnik und Schalldämpfer
- ☐ Gesetzliche Erlaubnis von Nachtzieltechnik und künstlichen Lichtquellen
- ☐ Jagd in befriedeten Gebieten bzw. Naturschutzgebieten zulassen
- ☐ Zahlung von angemessenen Prämien für erlegtes Schwarzwild
- ☐ Weniger bürokratischer Aufwand für den Erhalt von Prämien
- ☐ Mehr Sammelstellen / Lagerungsstätten für erlegtes Schwarzwild
- ☐ Förderung der Vermarktung und Verwertung von Schwarzwild-Wildbret
- ☐ Mehr Annahmestellen oder Unterstützung beim Versand von Probenmaterial (Blut-/Organproben zur ASP-Untersuchung)
- ☐ Andere Optionen
  Bitte teilen Sie uns mit, welche anderen Optionen Sie motivieren würden.
- ☐ Keine der genannten Optionen
  Bitte teilen Sie uns mit, warum keine der Optionen Sie motivieren würde.

Seite 10

17. Welche der genannten Optionen würde Sie mit hoher Wahrscheinlichkeit dazu motivieren, vermehrt nach Wildschweinkadavern zu suchen und diese zu beproben?

- [ ] Bereitstellung von Kadaver-Such-Hunden
- [ ] Weniger bürokratischer Aufwand bei der Meldung von Fallwild
- [ ] Zahlung von angemessenen Prämien für die Meldung von Fallwild
- [ ] Weniger bürokratischer Aufwand für den Erhalt von Prämien
- [ ] Mehr Sammelstellen / Lagerungsstätten für Fallwild
- [ ] Mehr Annahmestellen oder Unterstützung beim Versand von Probenmaterial (Blut-/Organproben zur ASP-Untersuchung)
- [ ] Andere Optionen
  Bitte teilen Sie uns mit, welche anderen Optionen Sie motivieren würden.
- [ ] Keine der genannten Optionen
  Bitte teilen Sie uns mit, warum keine der Optionen Sie motivieren würde.

Seite 11

Fast geschafft!

Vielen Dank, dass Sie bis hierhin alle Fragen beantwortet haben.
Zum Schluss benötigen wir noch einige demographische Angaben und Angaben zu Ihrer Jagdaktivität.

18. Bitte teilen Sie uns Ihr Geschlecht mit.

- ◯ Weiblich
- ◯ Männlich
- ◯ Divers

19. Bitte teilen Sie uns mit, zu welcher Altersgruppe Sie gehören.

- ◯ Unter 20 Jahre
- ◯ 20 bis 40 Jahre
- ◯ 41 bis 60 Jahre
- ◯ Über 60 Jahre

20. Über wie viele Jahre Jagderfahrung verfügen Sie?

- ◯ Unter 3 Jahre
- ◯ 3 bis 10 Jahre
- ◯ 11 bis 30 Jahre
- ◯ Über 30 Jahre

21. Haben Sie regelmäßigen Kontakt zu privaten oder gewerblichen Hausschweinhaltungen?

- ◯ Ja
- ◯ Nein

22. Bitte wählen Sie die zutreffende Angabe aus.

Ich bin...

- ☐ Eigenjagdbesitzer*in
- ☐ Jagdpächter*in
- ☐ Inhaber*in eines Begehungsscheins/Jagderlaubnisscheins
- ☐ Sonstiges: ______
- ☐ Keine Angabe

23. Welche Stückzahl Schwarzwild erlegen Sie durchschnittlich pro Jagdjahr?

______

24. Bitte teilen Sie uns mit, in welchem Bundesland bzw. in welchen Bundesländern Sie jagen.

Eine Mehrfachauswahl ist möglich.

- ☐ Baden-Württemberg
- ☐ Bayern
- ☐ Berlin
- ☐ Brandenburg
- ☐ Bremen
- ☐ Hamburg
- ☐ Hessen
- ☐ Mecklenburg-Vorpommern
- ☐ Niedersachsen
- ☐ Nordrhein-Westfalen
- ☐ Rheinland-Pfalz
- ☐ Saarland
- ☐ Sachsen
- ☐ Sachsen-Anhalt
- ☐ Schleswig-Holstein
- ☐ Thüringen

25. Bitte teilen Sie uns die Postleitzahl Ihres Haupt-Jagdgebietes mit.

______

26. Jagen Sie zusätzlich auch im europäischen Ausland?

- ◯ Ja
- ◯ Nein

Sie haben das Ende unseres Fragebogens erreicht – vielen herzlichen Dank für Ihre Zeit.

Möchten Sie uns noch etwas mitteilen?

Möchten Sie gern über die Ergebnisse der Studie informiert werden? Wenn ja, hinterlegen Sie bitte hier Ihre E-Mail-Adresse oder Ihre Anschrift (für den Fall, dass Sie eine postalische Zusendung wünschen).

Diese Kontaktdaten werden getrennt von Ihren anderen Angaben abgespeichert, es handelt sich nicht um die Erhebung personenbezogener Daten. Es werden keinerlei Hinweise auf das Interview gespeichert.

- [ ] Ja, ich möchte über die Ergebnisse der Studie informiert werden.

Im Rahmen dieser Studie sollen zukünftig auch Gruppendiskussionen mit Jäger*innen durchgeführt werden. Wenn Sie Interesse an der Teilnahme haben, hinterlegen Sie gern hier unverbindlich Ihre Kontaktdaten (zum Beispiel E-Mail Adresse, Anschrift oder Telefonnummer) um weitere Informationen zu erhalten.

Diese Kontaktdaten werden getrennt von Ihren anderen Angaben abgespeichert, es handelt sich nicht um die Erhebung personenbezogener Daten. Es werden keinerlei Hinweise auf das Interview gespeichert.

- [ ] Ja, ich möchte unverbindlich weitere Informationen zur Teilnahme an den Gruppendiskussionen erhalten.

Letzte Seite

Vielen Dank für Ihre Teilnahme!

Wir möchten uns ganz herzlich für Ihre Mithilfe bedanken.

Ihre Antworten wurden gespeichert, Sie können das Browser-Fenster nun schließen.

Möchten Sie in Zukunft an interessanten und spannenden Online-Befragungen teilnehmen?

Wir würden uns sehr freuen, wenn Sie Ihre E-Mail-Adresse für das SoSci Panel anmelden und damit wissenschaftliche Forschungsprojekte unterstützen.

E-Mail: ______ [Am Panel teilnehmen]

Die Teilnahme am SoSci Panel ist freiwillig, unverbindlich und kann jederzeit widerrufen werden.
Das SoSci Panel speichert Ihre E-Mail-Adresse nicht ohne Ihr Einverständnis, sendet Ihnen keine Werbung und gibt Ihre E-Mail-Adresse nicht an Dritte weiter.

Sie können das Browserfenster selbstverständlich auch schließen, ohne am SoSci Panel teilzunehmen.

Lisa Rogoll, Friedrich-Loeffler-Institut, Institut für Epidemiologie – 2022
