# Supplementary material for: African Swine Fever in Wild Boar: German Hunters’ Perception of Surveillance and Control—A Questionnaire Study
Source: Animals (Basel). 2023 Sep 5;13(18):2813. doi: 10.3390/ani13182813 (PMC10525383; doi:10.3390/ani13182813)
Supplement: Supplementary file 1 [file animals-13-02813-s001.zip › Supplementary_Document_S2.pdf]

Control of African swine fever in Germany

African swine fever (ASF) reached Germany in September 2020. Despite intensive efforts and the use of comprehensive control measures, it continues to spread in the German wild boar population.

In the fight against ASF in wild boar, local emergency services, such as hunters, play a crucial role: They know the local conditions particularly well and have many years of experience in dealing with wild boar. For this reason, we would like to gain an insight into your opinions, ideas and concerns as hunters with regard to the support of control measures against ASF in wild boar. With the results we would like to evaluate the existing strategies and develop recommendations for adjustments.

It will take approximately 15 minutes to complete the questionnaire. Please only participate in the survey if you are an active hunter. Note: The questions listed are all related to ASF in wild boar only.

By completing this questionnaire, you agree that the data collected may be used anonymously for research purposes and publications.

Thank you very much for your participation!

1. Do you agree to the following statement?

"The elimination of ASF in wild boar populations in Germany is possible."

☐ I agree. ☐ I disagree. ☐ I don't know.

Why do you agree or disagree? Please give reasons for your decision.

2. Do you agree to the following statement?

"Hunters play an ESSENTIAL role in ASF control in wild boar in Germany."

☐ I agree. ☐ I disagree. ☐ I don't know.

Why do you agree or disagree? Please give reasons for your decision.

- Page 3 –

3. How do you assess your level of knowledge about ASF?

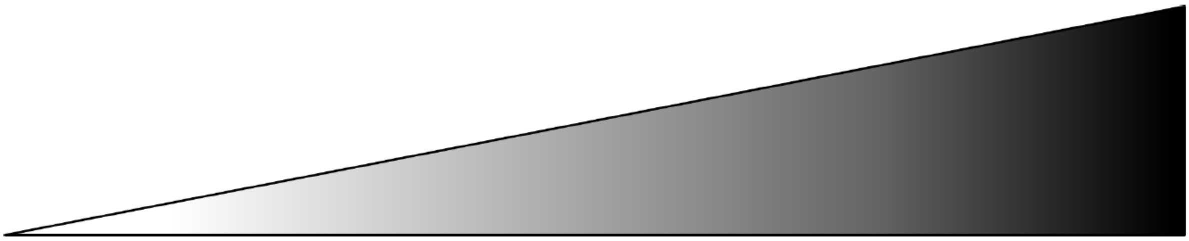

○	○	○	○	○
Very poor	Rather poor	Moderate	Rather good	Very good

4. From which sources do you draw your knowledge and your information about ASF?
Multiple selection is possible.

☐ magazines and newspapers for the hunting community
☐ written information from the hunting association
☐ written information from veterinary authorities
☐ written information of research and diagnostic institutes such as the Federal Research Institutes or state laboratories
☐ websites and internet forums for the hunting community
☐ websites of the hunting association
☐ websites of the veterinary authorities
☐ websites of research and diagnostic institutes
☐ social media such as Facebook, Instagram or Twitter
☐ television news or documentaries
☐ personal conversations with other hunters
☐ personal conversations with veterinary authorities
☐ events such as trade shows or lectures
☐ Other sources *(Please tell us from which other sources you draw your knowledge.)*

☐ No specification.

5. How satisfied were you with the cooperation with the respective mentioned institutions in the last 12 months?

	Very satisfied	Rather satisfied	Neutral	Rather unsatisfied	Very unsatisfied	No specification	No cooperation
Local hunting ring	o	o	o	o	o	o	o
State hunting association	o	o	o	o	o	o	o
National hunting association	o	o	o	o	o	o	o
Local agriculture	o	o	o	o	o	o	o
Local veterinary authority	o	o	o	o	o	o	o
The state laboratory	o	o	o	o	o	o	o
External forces (e.g. technical aid organizations, military or volunteers)	o	o	o	o	o	o	o

6. Do you feel yourself and your work valued by respective mentioned institutions?

	Yes	Rather yes	Neutral	Rather no	No	No specification	No cooperation
Local hunting ring	o	o	o	o	o	o	o
State hunting association	o	o	o	o	o	o	o
National hunting association	o	o	o	o	o	o	o
Local agriculture	o	o	o	o	o	o	o
Local veterinary authority	o	o	o	o	o	o	o
The state laboratory	o	o	o	o	o	o	o
External forces (e.g. technical aid organizations, military or volunteers)	o	o	o	o	o	o	o

7. Which consequences of ASF surveillance and control measures do you expect or experience?
Multiple selection is possible.

☐ Reduction of your own hunting activity (regarding single hunt)

☐ Increase of your own hunting activity (regarding single hunt)

☐ Local restriction of your own hunting activity

☐ Reduction of the wild boar population in your hunting district

☐ Increased personal work and time load

☐ Increase of personal costs

☐ Conflicts with veterinary authorities

☐ Conflicts with other hunters

☐ Conflicts with farmers

☐ Other consequences *(Please tell us which other consequences you expect or experience.)*

☐ No specification.

8. Which measures to prevent and control ASF have you implemented in the last 12 months?
Multiple selection is possible.

	ASF surveillance (in non-affected areas)	ASF control (in affected areas)	Not implemented
Sampling of hunted wild boar that appeared sick or wild boar found dead	o	o	o
Carcass search in the course of their usual hunting trips	o	o	o
Organised search for carcasses e.g. with human chains, search dogs or drones	o	o	o
Removal of wild boar carcasses	o	o	o
Increased hunting of wild boar	o	o	o
Increased hunting of adult females	o	o	o
Use of hunting traps	o	o	o
Construction of fences	o	o	o

9. Are there any additional measures to prevent and control ASF that you have implemented? If yes, please tell us which:

10. How do you assess the effectivity of respective measures regarding the successful control of ASF?

	Very effective	Fairly effective	Moderately effective	Hardly effective	Not effective	I don't know
Sampling of wild boar hunted or found dead	o	o	o	o	o	o
Intensive search for and removal of wild boar carcasses	o	o	o	o	o	o
Increased hunting of wild boar	o	o	o	o	o	o
Increased hunting of adult females	o	o	o	o	o	o
Increased hunting of young animals	o	o	o	o	o	o
Cleaning and disinfection of hunting equipment/clothing and vehicles	o	o	o	o	o	o
Use of wild boar traps	o	o	o	o	o	o
Construction of fences	o	o	o	o	o	o
Temporary ban on driven hunts after ASF outbreak	o	o	o	o	o	o
Permanent ban on driven hunts after ASF outbreak	o	o	o	o	o	o
Temporary ban on hide hunting after ASF outbreak,	o	o	o	o	o	o
Permanent ban on hide hunting after ASF outbreak,	o	o	o	o	o	o
Temporary ban on any hunting activity after ASF outbreak	o	o	o	o	o	o
Permanent ban on any hunting	o	o	o	o	o	o

11. Are there any additional measures, that have not been mentioned before, that you consider very effective? If yes, please tell us which:

12. How often do you go hunting for wild boar on average per month?

☐ Less than 1 time
☐ 1 to 5 times
☐ 6 to 10 times
☐ Over 10 times

13. Do you already use silencers or night vision devices for wild boar hunting?

☐ Night vision devices
☐ Silencers
☐ Both
☐ Neither

14. Would you be willing to operate wild boar traps and to cull wild boar in these traps in case of an ASF outbreak?

☐ Yes ☐ No

15. Which financial incentives regarding ASF surveillance and control can you claim in your Federal State?

☐ Incentives for hunted wild boar
☐ Incentives for notification of wild boar found dead
☐ Incentives for sampling of wild boar

☐ I don’t know.
☐ None.

16. Which of the suggested options would most likely motivate you to go hunting for wild boar more often to reduce the wild boar population?

☐ Provision of additional hunting tools such as night vision devices or silencers

☐ Extension of legal permission of night-time hunting devices ("*Nachtzieltechnik*")

☐ Permission of hunting in restricted areas and nature reserves

☐ Payment of appropriate financial rewards for hunted wild boar

☐ Reduction of bureaucracy in the process of receiving financial rewards

☐ Establishment of more collection points and storage facilities for hunted wild boar

☐ Establishment of more collection points for sample drop-off and support for shipping of sample materials (blood/organ samples) for ASF examination

☐ Promotion of marketing and use of wild boar meat and products

☐ Other options *(Please tell us which other options would motivate you.)*

☐ None *(Please tell us why none of the options would motivate you.)*

17. Which of the suggested options would most likely motivate you to go hunting for wild boar more often to reduce the wild boar population?

☐ Provision of carcass search dogs

☐ Reduction of bureaucratic effort for the notification of wild boar found dead

☐ Payment of appropriate financial rewards for notification of wild boar found dead

☐ Reduction of bureaucracy in the process of receiving financial rewards,

☐ Establishment of more collection points and storage facilities for wild boar carcasses

☐ Establishment of more collection points for sample drop-off and support for shipping of sample materials (blood/organ samples) for ASF examination

☐ Other options *(Please tell us which other options would motivate you.)*

☐ None *(Please tell us why none of the options would motivate you.)*

- Page 11 -

Almost done!

Thank you for answering all the questions up to this point. Finally, we need some demographic information and details about your hunting activity.

18. Please tell us your gender.

- ☐ Female
- ☐ Male
- ☐ Diverse

19. To which age group do you belong?

- ☐ Under 20 years
- ☐ 20 to 40 years
- ☐ 41 to 60 years
- ☐ Over 60 years

20. How many years of hunting experience do you have?

- ☐ Under 3 years
- ☐ 3 to 10 years
- ☐ 11 to 30 years
- ☐ Over 30 years

21. Do you have any regular contact with private or commercial pig farms?

☐ Yes ☐ No

22. Please select the applicable specification.

"I am… "

- ☐ An owner of a hunting district (*"Eigenjagdbesitzer"*)
- ☐ A lease-holder of a hunting district (*"Jagdpächter"*)
- ☐ Owner of a walk-in-certificate (*"Begehungsscheininhaber"*)
- ☐ Other: ______
- ☐ No specification.

23. What is your average annual hunting bag?

______

24. Please tell us in which Federal State(s) you hunt.
Multiple selection is possible

- ☐ Baden-Wurttemberg
- ☐ Bavaria
- ☐ Berlin
- ☐ Brandenburg
- ☐ Bremen
- ☐ Hamburg
- ☐ Hesse
- ☐ Mecklenburg-Western Pomerania
- ☐ Lower Saxony
- ☐ North Rhine-Westphalia
- ☐ Rhineland Palatinate
- ☐ Saarland
- ☐ Saxony
- ☐ Saxony-Anhalt
- ☐ Schleswig-Holstein
- ☐ Thuringia

25. Please tell us the postal code of your main hunting area.

______

26. Do you as well hunt in other European countries?

- ☐ Yes
- ☐ No

- Page 13 -

You have reached the end of the questionnaire. Thank you very much for your time.

Is there anything else you would like to tell us?

Do you wish to be informed about the results of the study? If yes, please provide your e-mail address or your address in case you wish a postal information.

Your contact details will be saved separately from your answers – we do not collect personal data in this interview. No conclusions about your interview can be drawn.

☐ Yes, I wish to be informed about the results of the study.

It is planned to conduct additional focus group discussions with hunters. If you are interested in participating, please provide your contact details (e-mail address, address or phone number) to receive further non-binding information.

Your contact details will be saved separately from your answers – we do not collect personal data in this interview. No conclusions about your interview can be drawn.

☐ Yes, I wish to be informed non-bindingly about participation in focus group discussions.
